# Computational design and engineering of self-assembling multivalent microproteins with therapeutic potential against SARS-CoV-2

**DOI:** 10.1186/s12951-024-02329-3

**Published:** 2024-02-10

**Authors:** Qin Qin, Xinyi Jiang, Liyun Huo, Jiaqiang Qian, Hongyuan Yu, Haixia Zhu, Wenhao Du, Yuhui Cao, Xing Zhang, Qiang Huang

**Affiliations:** 1 State Key Laboratory of Genetic Engineering, Shanghai Engineering Research Center of Industrial Microorganisms, MOE Engineering Research Center of Gene Technology, School of Life Sciences, Fudan University, Shanghai, 200438 China; 2 ACROBiosystems Inc, Beijing, 100176 China; 3 Multiscale Research Institute of Complex Systems, Fudan University, Shanghai, 201203 China

**Keywords:** SARS-CoV-2, Protein therapeutics, Microprotein, Nanobody, Computational design, Cryo-EM

## Abstract

Multivalent drugs targeting homo-oligomeric viral surface proteins, such as the SARS-CoV-2 trimeric spike (S) protein, have the potential to elicit more potent and broad-spectrum therapeutic responses than monovalent drugs by synergistically engaging multiple binding sites on viral targets. However, rational design and engineering of nanoscale multivalent protein drugs are still lacking. Here, we developed a computational approach to engineer self-assembling trivalent microproteins that simultaneously bind to the three receptor binding domains (RBDs) of the S protein. This approach involves four steps: structure-guided linker design, molecular simulation evaluation of self-assembly, experimental validation of self-assembly state, and functional testing. Using this approach, we first designed trivalent constructs of the microprotein miniACE2 (MP) with different trimerization scaffolds and linkers, and found that one of the constructs (MP-5ff) showed high trimerization efficiency, good conformational homogeneity, and strong antiviral neutralizing activity. With its trimerization unit (5ff), we then engineered a trivalent nanobody (Tr67) that exhibited potent and broad neutralizing activity against the dominant Omicron variants, including XBB.1 and XBB.1.5. Cryo-EM complex structure confirmed that Tr67 stably binds to all three RBDs of the Omicron S protein in a synergistic form, locking them in the “3-RBD-up” conformation that could block human receptor (ACE2) binding and potentially facilitate immune clearance. Therefore, our approach provides an effective strategy for engineering potent protein drugs against SARS-CoV-2 and other deadly coronaviruses.

**Graphical Abstract:**

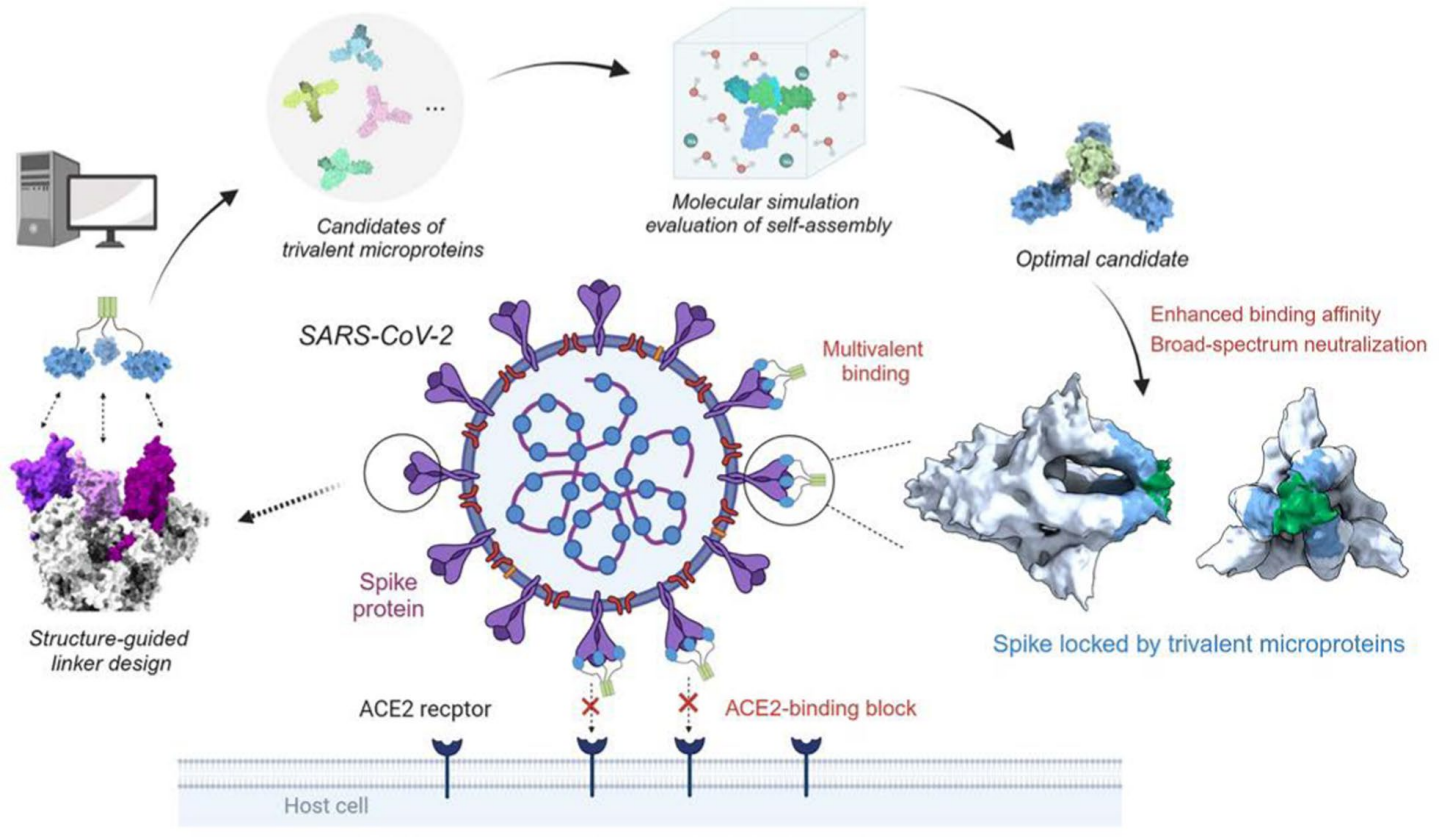

**Supplementary Information:**

The online version contains supplementary material available at 10.1186/s12951-024-02329-3.

## Introduction

The COVID-19 pandemic in the past 3 years has posed an enormous threat to human health, and will continue to do so as SARS-CoV-2 evolves to evade the humoral immunity elicited by vaccination or prior infection [1, 2]. Therefore, the development of highly effective drugs to treat COVID-19 remains critical. However, the ongoing mutations in SARS-CoV-2 not only diminish the efficacy of existing drugs, but also reduce the number of conserved epitopes in drug targets, posing a significant challenge to drug design and development [3, 4]. For example, the Omicron (B.1.1.529) variant, which bears 37 amino acid mutations in its glycosylated spike (S) protein that overlap many antibody epitopes, can escape the immune system and has rapidly become the dominant strain worldwide since its first identification in South Africa in November 2021 [5]. It has been reported that 85% of the 247 antibodies that directly target the S protein fail to neutralize the Omicron variants [6]. Given this situation, there is an urgent need to develop novel therapeutic agents that can effectively combat emerging SARS-CoV-2 variants, such as broad-spectrum neutralizing antibodies or other protein drugs.

As mentioned, the S protein plays a critical role in viral infection by facilitating viral entry into host cells. This protein is a homotrimer on the viral membrane, and each monomer consists of the receptor-binding subunit S1 and the membrane-fusion subunit S2 [7]. During viral infection, the receptor-binding domains (RBDs) at the top of the S1 subunits interact directly with the host cell receptor angiotensin-converting enzyme 2 (ACE2) [8]. This interaction triggers a series of conformational changes in the S protein that lead to fusion of the viral and cellular membranes, ultimately delivering the viral genome into the host cells [9]. Therefore, RBD is a major target for neutralizing antibodies that can block the binding of ACE2 to the S protein. The trimeric RBDs make the S protein an ideal target for the multivalent drugs. In principle, multivalent drugs can use their pharmacophores (e.g., an antibody to a single RBD) to synergistically bind to the three RBDs of the S protein, thereby enhancing therapeutic effectiveness against the viruses [10–13].

In the past, nanoscale multivalent proteins have been developed to target the S protein or other disease proteins with homo-oligomeric binding sites. The most common method is to link monovalent binders, such as nanobodies, in a head-to-tail tandem fashion with fusion linkers, resulting in enhanced binding affinities and neutralizing activities compared to the monovalent counterparts [11, 14–17]. Because the tandem nanobodies might produce elongated structures that can reduce protein stability and increase susceptibility to degradation, other multivalent formats of the proteins have also been constructed. For instance, based on the trimeric structure of the S protein, previous studies have reported self-assembling trivalent ACE2 that can effectively neutralize SARS-CoV-2 [18–20]. However, the large size (~ 615 aa) and poor solubility of ACE2 in engineered bacteria raise concerns about manufacture and steric hindrance that could lead to unwanted interactions with non-target proteins and adverse side effects [21].

To address these issues, the use of new multivalent formats and soluble, easily produced nanoscale microproteins (less than 150–200 aa) [22] as the monovalent binders is helpful. Strauch et al. have developed trimeric influenza-neutralizing proteins that target the three receptor binding sites of influenza hemagglutinin (HA) using a trimerization domain identified from the PDB [23]. Similarly, Cui et al. constructed a potent TNF-α antagonist by fusing a soluble receptor TNFRII to a trimerization domain from human type III collagen [24]; Chen et al. fused protein of gp41 NHR to the T4 fibritin trimerization domain to construct trimeric anti-HIV-1 therapeutics [25]. These results suggest that multivalent proteins with soluble microproteins and a self-assembling trimerization scaffold may also be suitable for targeting the homo-trimeric binding sites of the S protein. However, except for the study by Strauch et al. [23], many multivalent designs were tested by trial and error, and usually were time-consuming and resource-intensive. Therefore, it remains of great interest to develop effective rational design methods to construct nanoscale multivalent proteins targeting the S protein.

**Fig. 1:**
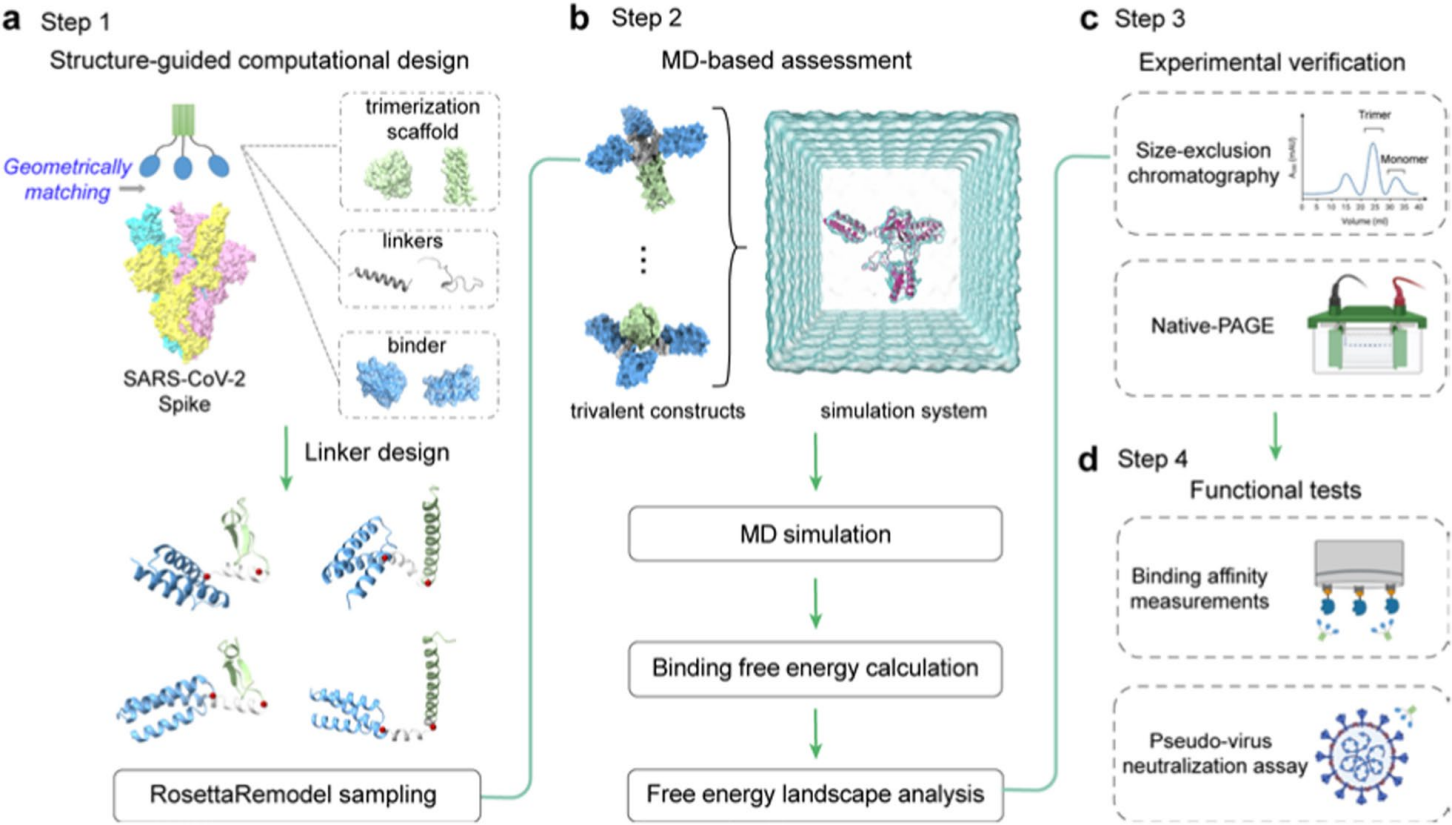
Workflow for computational design of trivalent anti-SARS-CoV-2 microproteins. **a** Structure-guided computational design of trivalent microproteins to geometrically match the three binding sites of the trimeric S protein. The trimerization scaffold, linker, and monovalent binder are shown in blue, green, and gray, respectively. RosettaRemodel was used to design linkers connecting the C-terminus of the monovalent binder and the N-terminus of the trimerization scaffold. **b** Molecular dynamics (MD) evaluation of the trivalent constructs. Binding free energies of the monomers were estimated to assess trimerization tendency using the MM/GBSA method. Free energy landscapes were constructed to study the possible distributions of the trimer conformations. **c** Experimental verification of trivalent constructs using size-exclusion chromatography and Native-PAGE. **d** Functional tests of the top-ranked constructs. Binding affinity was measured by BLI, and pseudovirus neutralization assays were performed to determine the neutralizing activity against SARS-CoV-2

Here, we present a computational approach to design and engineer self-assembling trivalent microproteins targeting the S protein of SARS-CoV-2. As illustrated in Fig. 1, this approach involves four steps: structure-guided linker design, molecular simulation evaluation of self-assembly, experimental validation of self-assembly state, and functional testing. Using this approach, we successfully engineered two trivalent proteins with high antiviral potency against SARS-CoV-2: MP-5ff and Tr67, using the microprotein miniACE2 and the nanobody Nb67 as the monovalent binders, respectively. Both multivalent proteins exhibited efficient self-assembling trimerization and good conformational homogeneity. As expected, they showed significantly higher binding affinities and neutralizing activities than the monovalent counterparts. Moreover, Tr67 was shown to be effective against dominant Omicron variants, including XBB.1 and XBB.1.5. Cryo-electron microscopy (cryo-EM) analysis confirmed that Tr67 indeed binds to the Omicron S protein in a trivalent mode and induces it into the unique “3-RBD-up” conformation.

## Results

### Structure-guided linker design

**Fig. 2:**
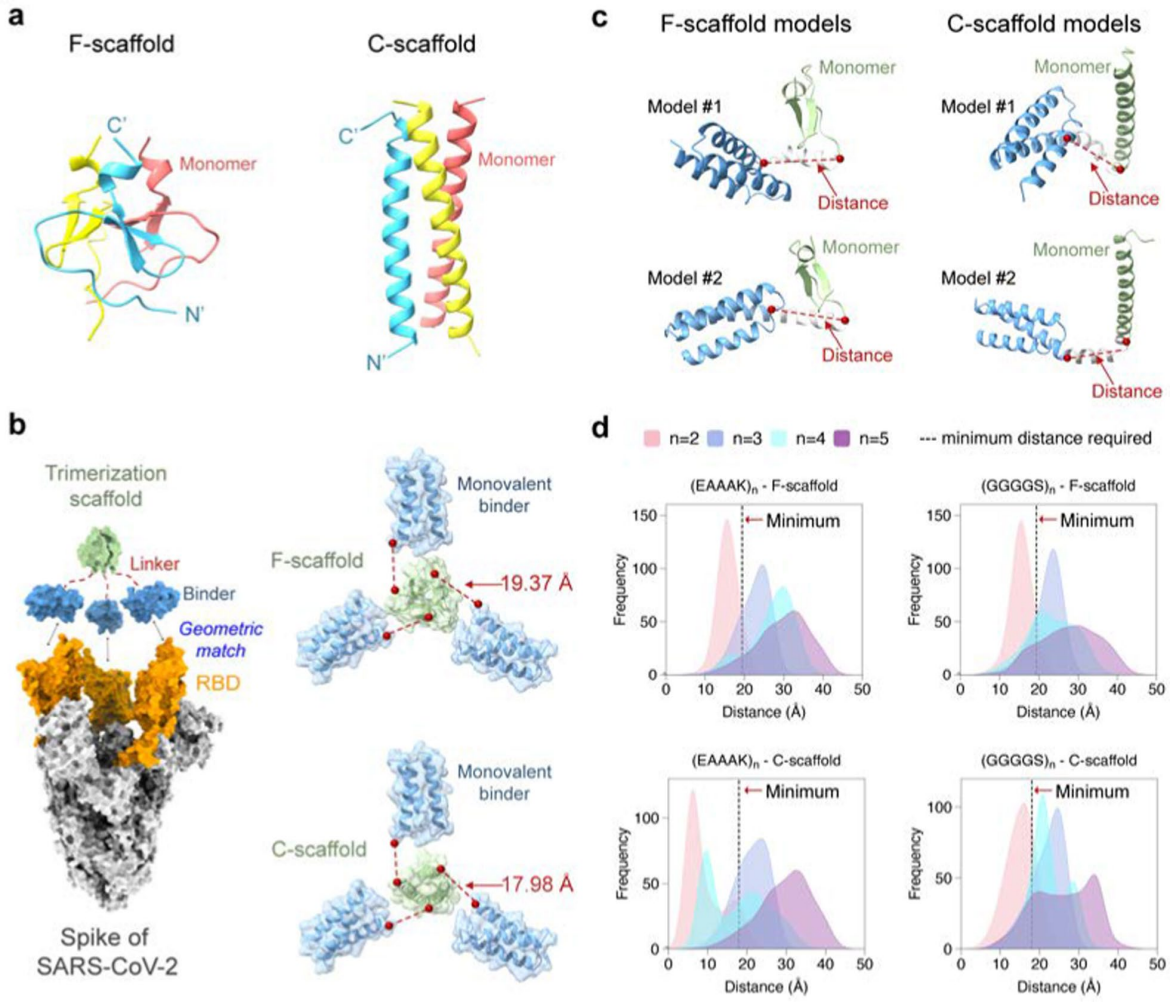
Structure-guided linker design. **a** Two trimerization scaffolds used in this study. **b** The left panel: The trivalent construct designed to match the geometry of the three binding sites on RBDs (orange) of the S protein (silver). The monovalent binder and the trimerization scaffold are shown in blue and green, respectively. The right panel: The calculated minimum distances required between the binder and scaffold. **c** Schematic of linker models generated by RosettaRemodel. For each model, the distance from the C-terminus of the binder to the N-terminus of the scaffold was determined. **d** Distributions of the mentioned distances of the designed linkers of different lengths. The dotted black lines indicate the minimum distances required for the constructs

To design a self-assembling trivalent protein with desired physiochemical properties and therapeutic efficacy, we carefully selected two essential components: the monovalent therapeutic agent and the trimerization scaffold. As mentioned above, microproteins are well suited to be engineered into multivalent formats due to their small size, stability, and ease of production. As a proof of concept, we first used the microprotein LCB3 [26] as a monovalent binder to the S protein of SARS-CoV-2. This microprotein (MP) is a mini-mimetic of the ACE2 protein with only 64 aa (hereafter we refer to it as miniACE2) and has been reported to bind to the RBD of the S protein. To obtain optimal multivalent constructs, we then selected two well-studied self-assembling domains as the test trimerization scaffolds, namely the β-propeller-like foldon domain of T4 fibritin used in vaccines [27–29] and an α-helical coiled-coil peptide [30], which will be referred to as F-scaffold and C-scaffold, respectively (Fig. 2a).

We hypothesized that a well-designed trivalent protein could simultaneously engage all three RBDs of the S protein, thereby blocking the ACE2 binding and enhancing its neutralizing activity against the SARS-CoV-2 variants. It has been shown that RBD can adopt two different conformations: standing-up conformation (RBD-up) for receptor binding and lying-down conformation (RBD-down) for immune evasion [7, 31, 32]. The RBD-up state is essential for membrane fusion and virus entry [9, 31], and potentially facilitates immune clearance. Therefore, our designed goal was to trap the active RBD-up conformation by fully occupying all three RBDs with a designed trivalent protein. To achieve this, we superimposed the miniACE2-RBD complex (PDB ID: 7JZM) onto the S protein with the 3-RBD-up state (PDB ID: 7CT5). Based on the superimposed structure, we calculated the minimum distance required for a linker to connect miniACE2 and the given trimerization scaffold using the Lagrange multiplier method, and found that the minimum distances for the F- and C-scaffolds are 19.37 Å and 17.98 Å, respectively.

Based on the minimum distances, we then designed linkers to connect the monovalent binder and the trimerization scaffold, ensuring an appropriate geometry to match the homo-trimeric target sites. We selected two widely used penta-peptide fragments, the flexible GGGGS and the rigid EAAAK [33], as the building fragment for the candidate linkers, and then determined the repeat number (*n*) of the given fragment in the linker according to the minimum distances and the folding conformations of each linker. Considering the maximum length of the extended conformation of a penta-peptide (GGGGS or EAAAK), at least two copies of the fragments (i.e., 10-aa length) are needed for a linker to connect the binder to the scaffold. To determine the optimal repeat number *n*, we used RosettaRemodel to sample a large number of the lowest-energy folding conformations for the linkers of (GGGGS)_n_ or (EAAAK)_n_ (*n* = 2, 3, 4, or 5) (see Materials and methods). For each *n*, the folding conformations of the given linker were predicted with the binder at its N-terminus and the scaffold at the C-terminus; and in the calculations, both the binder and the scaffold were treated as rigid bodies. After the conformational sampling, the 1000 top-ranking lowest-energy conformations were used to calculate the distributions of the distances between the C-terminus of the binder and the N-terminus of the scaffold (Fig. 2).

As shown in Fig. 2b, most of the sampling distances for the linkers (GGGGS)_2_ and (EAAAK)_2_ fell short of the above-mentioned minimum distances required for the geometric matching of the binder and the trimerization scaffold, indicating that linkers designed with *n* = 2 may not be suitable. In contrast, most distances for the linkers (GGGGS)_n_ and (EAAAK)_n_, with *n* = 3, 4, or 5, exceeded the minimum distances, indicating that *n* ≥ 3 is required. Among these, the distance distributions for* n* = 3 were relatively narrow, while those for *n* = 4 or 5 were wider, indicating that more conformations were energetically possible for these linkers and thus that the binders connected to the trimerization unit could bind to more positions in space, allowing them to adapt their conformations to different epitopes on the target protein. However, the distance distributions for* n* = 4 were irregular, neither as narrow as *n* = 3 nor as broad as *n* = 5; furthermore, those for *n* = 3 and 5 have covered most of the range seen in *n* = 4, making n = 4 less preferable. Also, given that n = 5 already covered the possible distances, we no longer explored the situation of n > 5.

Finally, we chose four test linkers, the flexible (GGGGS)_3_ and (GGGGS)_5_, and the rigid (EAAAK)_3_ and (EAAAK)_5_, to construct the trivalent proteins for miniACE2, resulting in eight trivalent proteins tailored for the two trimerization scaffolds. We designated the two proteins using flexible (GGGGS)_n_ and C-scaffold as MP-3fc, MP-5fc, respectively; those using (GGGGS)_n_ and F-scaffold as MP-3ff, MP-5ff, respectively; those using rigid (EAAAK)_n_ and C-scaffold as MP-3rc, MP-5c, respectively; and those using (EAAAK)_n_ and F-scaffold as MP-3rf, MP-5rf, respectively (Additional file 1: Table S1).

### Molecular simulation evaluation of trivalent constructs

Biomedical and therapeutic applications of multivalent proteins usually require them to have good physicochemical properties such as efficient self-assembly and good conformational homogeneity. To identify the best candidate among the eight constructs, we evaluated these properties of the eight constructs using molecular dynamics (MD) simulations. For each construct, three independent simulations were performed, each with a simulation time of 300 ns. Then, we calculated the root mean square deviation (RMSD) of the protein backbone heavy atoms across the simulation trajectories, using their initial structures as the reference conformations (Additional file 1: Fig. S1). As can be seen in (Additional file 1: Fig. S1, the RMSD results showed that all the systems reached equilibrium after about 150-ns simulations. Therefore, we used the post-150 ns trajectories for the following analyses.

**Fig. 3:**
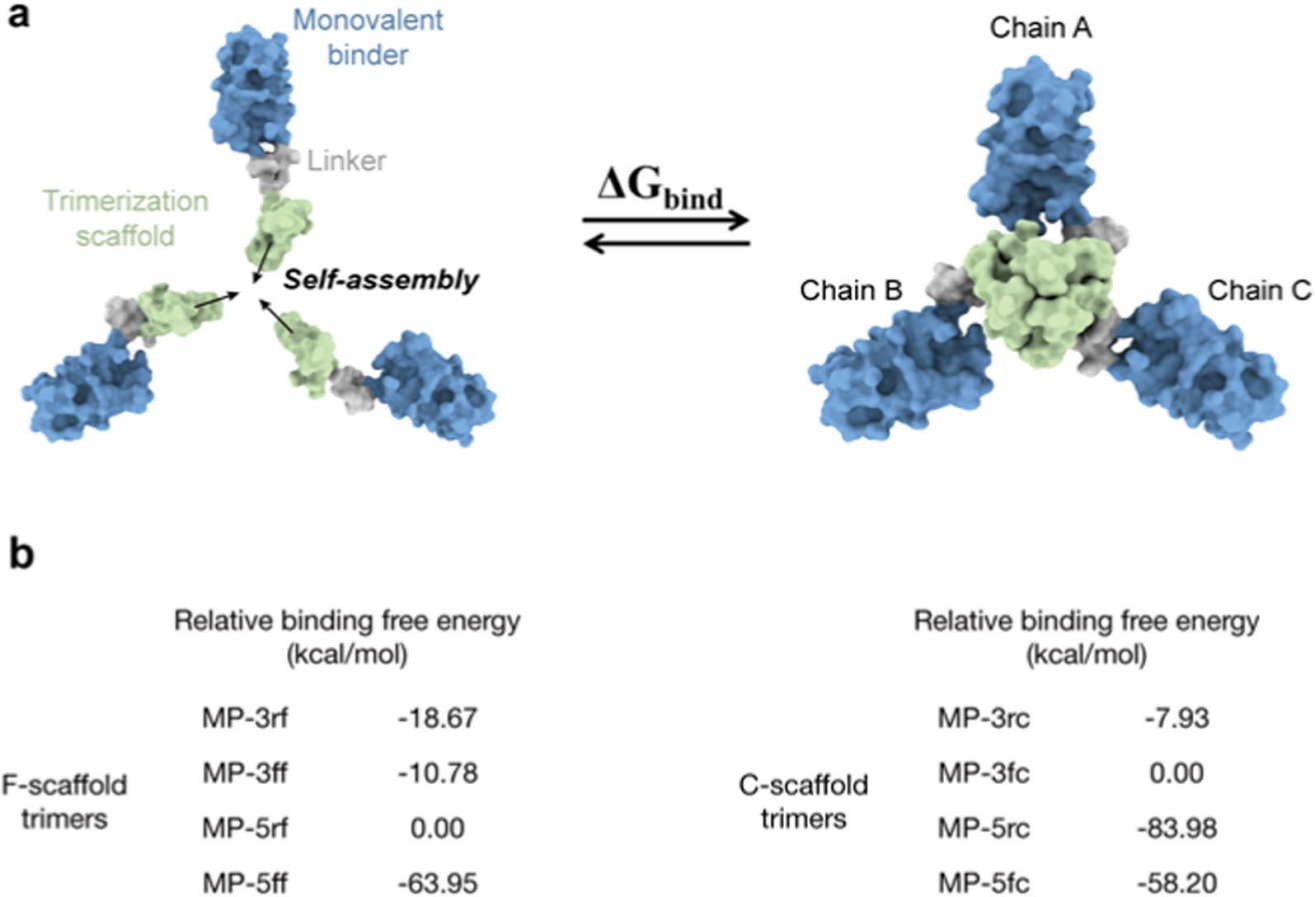
Molecular simulation assessment of the trimerization tendency of trivalent constructs. **a** Schematic diagram for the binding process to calculate the binding free energy of a given trivalent construct. **b** Relative binding free energies of the trivalent constructs calculated by MM/GBSA. For F-scaffold trimers, MP-5rf was used as the reference to calculate the relative values of other constructs. For C-scaffold trimers, the reference is MP-3rc. Please see the MM/GBSA raw data in (Additional file 1: Table S2

To assess the self-assembly abilities, we first used the Molecular Mechanics/Generalized Born Surface Area (MM/GBSA) method to estimate the binding free energies between the three monomers of the trivalent constructs, as illustrated in Fig. 3a and (Additional file 1: Table. S2). Although MM/GBSA has limitations in predicting absolute values of binding free energy, it excels in ranking the relative binding affinities of different molecules [34]. Similarly, the relative binding free energies of different constructs could also rank their self-assembly abilities. The MM/GBSA calculations showed that the binding free energies of the F-scaffold constructs are typically lower than those of the C-scaffold constructs, except for that of MP-5rc (Additional file 1: Table S2). This suggests that the self-assembly abilities of F-scaffold constructs are relatively better than those of the C-scaffold constructs. Among the F-scaffold constructs, the binding free energy of MP-5rf is the highest; as shown in Fig. 3b, the relative binding free energies of the other three constructs are negative, indicating that their self-assembly abilities are stronger than MP-5rf. Of them, MP-5ff has the lowest relative binding free energy, implying the strongest self-assembly tendency. For the C-scaffold constructs, MP-5rc has the lowest relative binding free energy, indicating a stronger self-assembly ability, especially compared with the higher relative energy values of MP-3fc and MP-3rc.

**Fig. 4:**
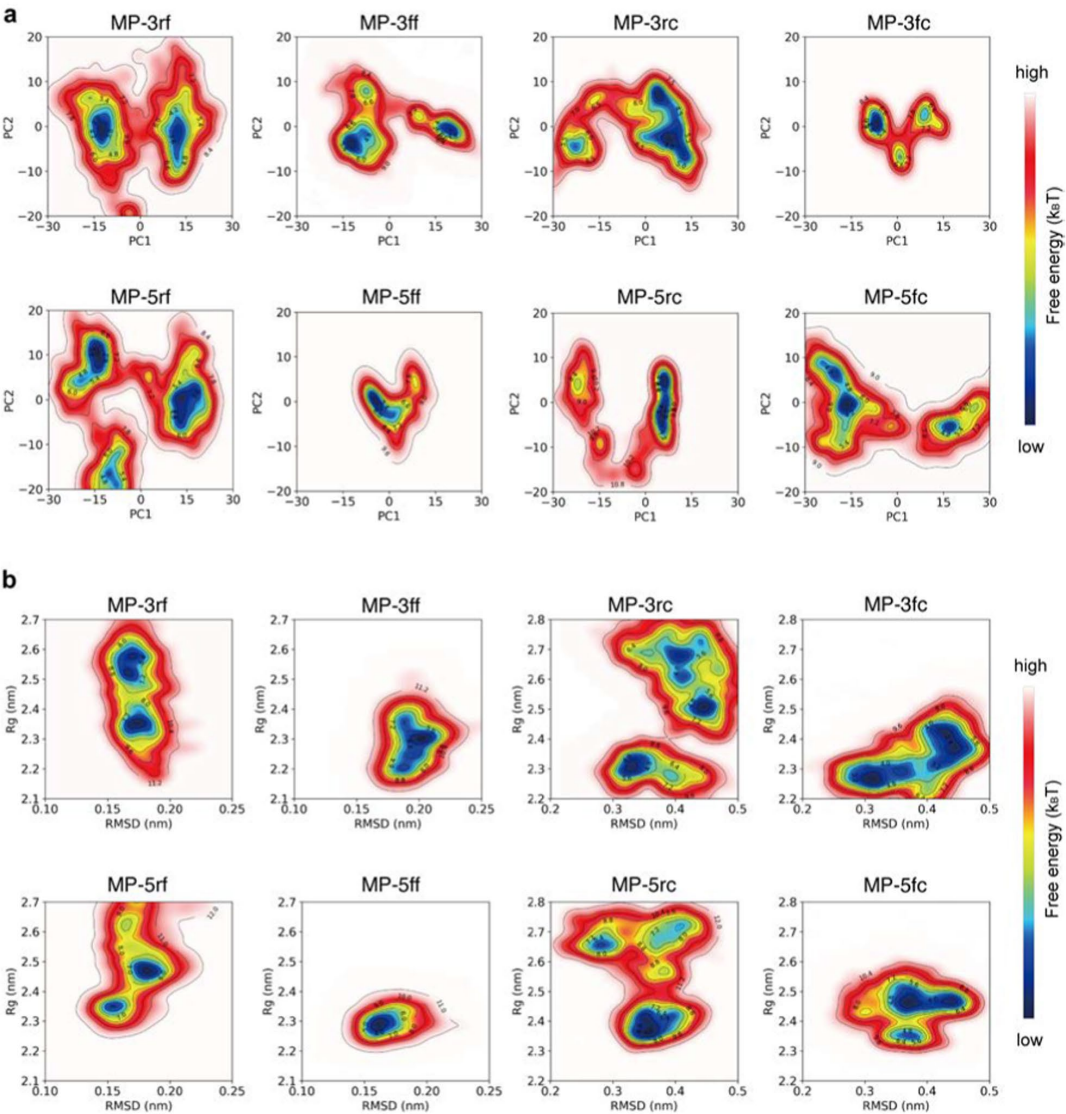
Free energy landscapes (FELs) for the MD conformations of the trivalent constructs. **a** FELs of conformational projections onto the first and the second principal components (PC1 and PC2). **b** FELs of conformational projections onto two alternative reaction coordinates: root mean square deviation (RMSD) and radius of gyration (Rg)

To investigate the conformational homogeneity, we first performed principal component analysis (PCA) on the simulation trajectories and then mapped the simulated conformations of the proteins onto the resulting principal components to generate their free energy landscapes (FELs) (see Methods and materials). As an example, the PCA results for a trajectory of MP-5ff are shown in (Additional file 1: Fig. S2). As seen, the first two principal components contributed to over 80% of the cumulative variance and were thus considered PC1 and PC2 (Additional file 1: Figs. S2A, D). By projecting the simulated conformations onto the two-dimensional space of PC1 and PC2, the resulting FELs showed the distribution patterns of the simulated constructs and their possible numbers of dominant trimer-like conformations in the simulations (Fig. 4).

As shown in Fig. 4a, the FEL patterns of the eight constructs are not identical, with about 1–3 low-energy wells (in blue) indicating different numbers of dominant trimer-like conformations in the simulations. Significantly, except for MP-5ff, other constructs had a wider or more than one low-energy well, such as MP-5rc with a wider low-energy well, MP-3rf, MP-5rf with 2 low-energy wells, and MP-3ff, MP-3fc, MP-5fc with 3 low-energy wells. Thus, only MP-5ff showed only a low-energy trimer conformation in the simulations, suggesting that the conformational homogeneity of this protein is the best.

To further confirm the FEL results, we also construct FELs by mapping the simulated conformations onto the two-dimensional space defined by two alternative reaction coordinates: root mean square deviation (RMSD) and radius of gyration (Rg) (Fig. 4b). Consistent with the results of Fig. 4a, except MP-5ff the FELs of all constructs had multiple low-energy wells (in blue), suggesting that multiple low-energy trimer-like conformations coexist in the simulations. Taken together, the results in Fig. 4 suggested that MP-5ff likely has a single stable trimer conformation and thus the best conformational homogeneity.

### Experimental validation and functional test

**Fig. 5:**
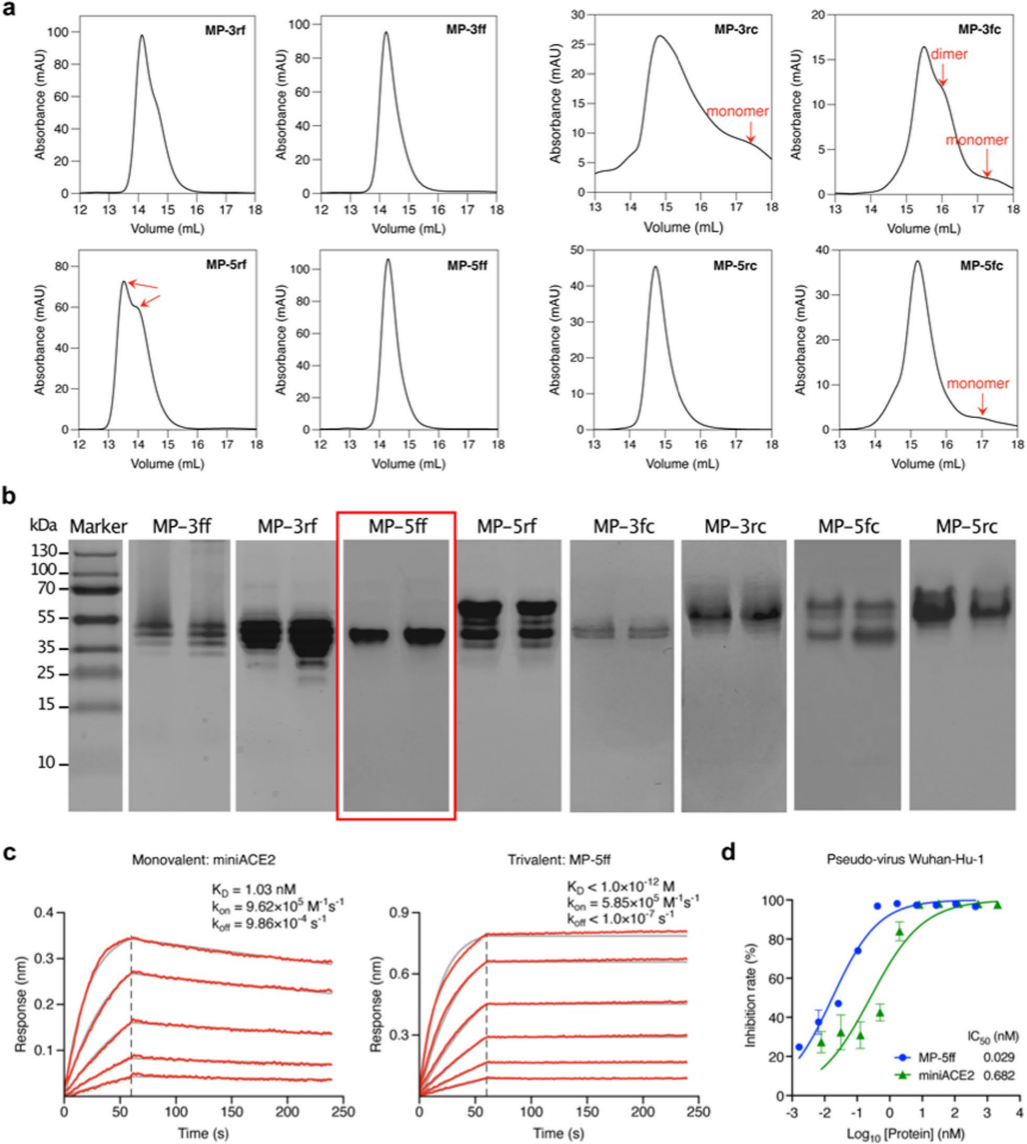
Experimental verifications of the trivalent constructs of miniACE2. **a** SEC profiles of the purified proteins of the constructs. **b** Native-PAGE analysis of the protein trimer fractions isolated from SEC. **c** BLI measurements of the binding kinetics of the monovalent miniACE2 and the trivalent construct, MP-5ff, to the immobilized RBD of SARS-CoV-2 (Wuhan-Hu-1). Red traces represent the raw data and the kinetic fits are shown in gray. **d** Neutralizing activity of miniACE2 and MP-5ff against SARS-CoV-2 pseudovirus (Wuhan-Hu-1)

To validate the computational results, we expressed the 8 designed constructs in *E. coli* Rosetta (DE3) cells and purified the proteins using Ni–NTA affinity chromatography. We then characterized their oligomeric states in solution by size-exclusion chromatography (SEC). As shown in Fig. 5a, the SEC profiles revealed that the four F-scaffold proteins had narrower and sharper trimer peaks than the C-scaffold proteins, indicating a higher trimer ratio in the F-scaffold constructs. Among the F-scaffolds, the peak of MP-5ff appears to be the sharpest and the most concentrated one, indicating that it is the most efficient trivalent construct, in good agreement with the computational evaluation. In contrast, MP-5rf displayed two distinct peaks, probably corresponding to the desired trimerization conformation and another oligomeric state. Indeed, the binding free energy calculations in Fig. 3b have already indicated that the MP-5rf trivalent construct is less stable than the other three F-scaffold constructs. As for the C-scaffold constructs, only MP-5rc had a sharp, single peak indicating a trimer; however, besides the trimer peak, the other three constructs had detectable monomer or dimer peaks, especially MP-3rc and MP-3fc, suggesting that they had a lower trimer ratio. Obviously, these results confirmed the MM/GBSA calculations (Fig. 3b) and showed that those constructs with the lower binding free energies have higher trimerization efficiencies.

The FEL analyses in the last subsection have suggested that even in the trimer state, the investigated trivalent constructs are likely to contain several different trimer-like conformations (Fig. 4). To further investigate the possible distributions of trimerization conformations, we performed Native-PAGE analysis on the protein samples collected from the SEC trimer peaks, because this technique can separate two or more trimer-like conformations of the trivalent proteins. As shown in Fig. 5b, among the eight constructs, only MP-5ff presented a single protein band, indicating a single stable trimer conformation, which is consistent with the computational prediction showing only a single energy well in the FELs (Figs. 4a, b). In contrast, MP-3ff and MP-3rf showed multiple distinct bands, indicating that they can adopt several coexisting conformations. For MP-5rf, we observed two dominant bands with several fainter ones at various positions; the upper one may suggest the formation of larger oligomers that fail to maintain a stable trimer. Similarly, MP-5fc also displayed such a pattern. For the other three C-scaffold constructs, we also observed more than one band: MP-3fc exhibited two clear bands, while MP-3rc and MP-5rc showed a clear one and several fainter bands. These findings validate the computational predictions that several low-energy trimer-like conformations may coexist for these constructs (Figs. 4a, b). As a result, MP-5ff was found to be the best construct with the highest trimerization efficiency and conformational homogeneity.

We next examined the target binding affinity of the optimal construct, MP-5ff, to RBD of the S protein using Biolayer interferometry (BLI). Since miniACE2 was originally designed to specifically target the SARS-CoV-2 Wuhan-Hu-1 strain [26], here we focused our functional evaluation on miniACE2 and MP-5ff against this specific strain. As shown in Fig. 5c, MP-5ff exhibited a much slower dissociation rate (*k*_off_ < 1.0 × 10^–7^ s^−1^) compared to that of miniACE2 (*k*_off_ = 9.86 × 10^–4^ s^−1^); thus, the resulting equilibrium dissociation constant (K_D_) is less than 1 pM, while that of miniACE2 is 1.03 nM. Thus, the binding affinity of MP-5ff for RBD is 1000-fold greater than that of its monovalent counterpart miniACE2, clearly demonstrating that protein multivalency could substantially enhance the target binding affinity. Then, we evaluated the neutralizing activities of miniACE2 and MP-5ff against SARS-CoV-2 pseudovirus (Wuhan-Hu-1). As indicated in Fig. 5d, the monovalent miniACE2 was already able to inhibit the virus with an IC_50_ of 682 pM; nonetheless, the trivalent MP-5ff still significantly enhanced the neutralizing activity (IC_50_ = 29 pM), exhibiting a 23-fold increase. Taken together, the trivalent MP-5ff designed by our rational approach has excellent physicochemical properties and potent antiviral activity.

### Engineering of a broad-spectrum trivalent nanobody

**Fig. 6:**
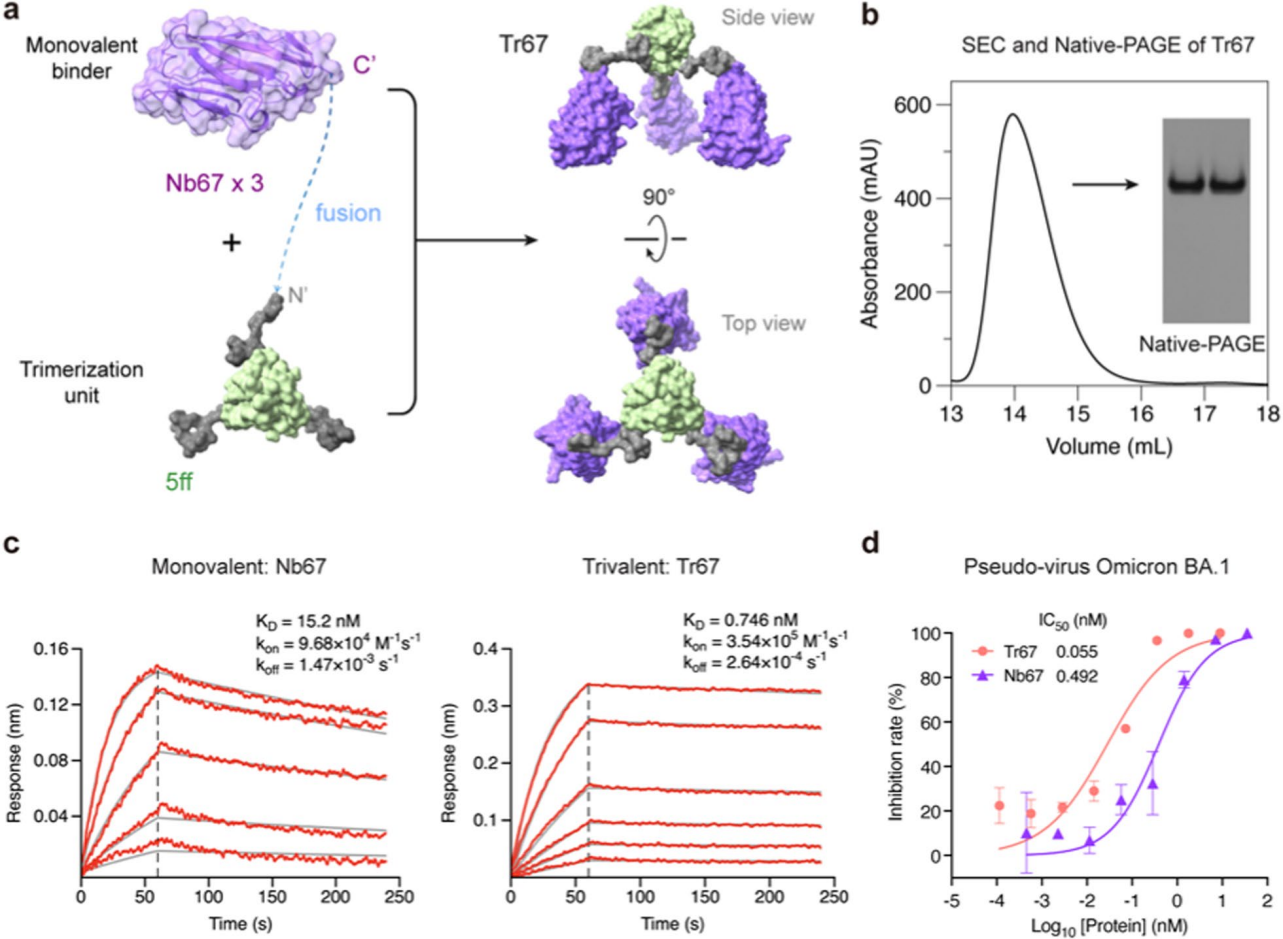
Design and experimental characterization of Tr67. **a** Schematic diagram illustrating the trimerization of Nb67 nanobody fused with the optimal trimerization unit 5ff (see its amino-acid sequence of the fusion monomer of Nb67 with 5ff in (Additional file 1: Table S1). **b** SEC and Native-PAGE analysis of Tr67, showing high trimerization tendency and conformational homogeneity. **c** BLI measurement of the binding kinetics of the monovalent Nb67 and Tr67 to the immobilized RBD of SARS-CoV-2 pseudovirus (Omicron BA.1). **d** Neutralizing activities of Nb67 and Tr67 against SARS-CoV-2 pseudovirus (Omicron BA.1). Three independent experiments were performed

To further demonstrate the effectiveness of our approach, we applied the 5ff trimerization unit to engineer a trivalent nanobody targeting the dominant circulating Omicron variants, because nanobodies represent another widely used category of microproteins well suited for multivalent construction. For this purpose, we selected Nb67, a nanobody identified by Xiang et al. [35] from serially immunized camelid sera, which was reported to neutralize Omicron BA.1. By fusing Nb67 with the 5ff trimerization unit, we created a trivalent nanobody Tr67 (Fig. 6a, (Additional file 1: Table S1). Following the same computational and experimental procedures successfully employed for MP-5ff, we assessed the trimerization efficiency and conformational homogeneity of the engineered Tr67 using MD simulations ((Additional file 1: Fig. S3), SEC and native-PAGE analyses (Fig. 6b). These obtained results demonstrated that Tr67 has a trimerization efficiency and conformational homogeneity very similar to that of MP-5ff.

We then measured the binding affinity of Tr67 to the target RBD of the S protein using BLI and its neutralizing activity against SARS-CoV-2 pseudoviruses. As shown in Fig. 6c, Tr67 exhibited a higher association rate (k_on_) and a lower dissociation rate (k_off_) compared to its monovalent counterpart Nb67. The resulting K_D_ was 0.746 nM, which is an about 20-fold increase in affinity compared to that of the monovalent Nb67 (K_D_ = 15.2 nM). Similarly, Tr67 showed much stronger inhibitory activity against the SARS-CoV-2 Omicron BA.1 pseudovirus than Nb67, with an IC_50_ of 55 pM versus 492 pM for Nb67 (Fig. 6d). These results demonstrated that the trivalent nanobody has an enhanced potency and thus greater potential to combat viral infections compared to its monovalent counterpart.

**Fig. 7:**
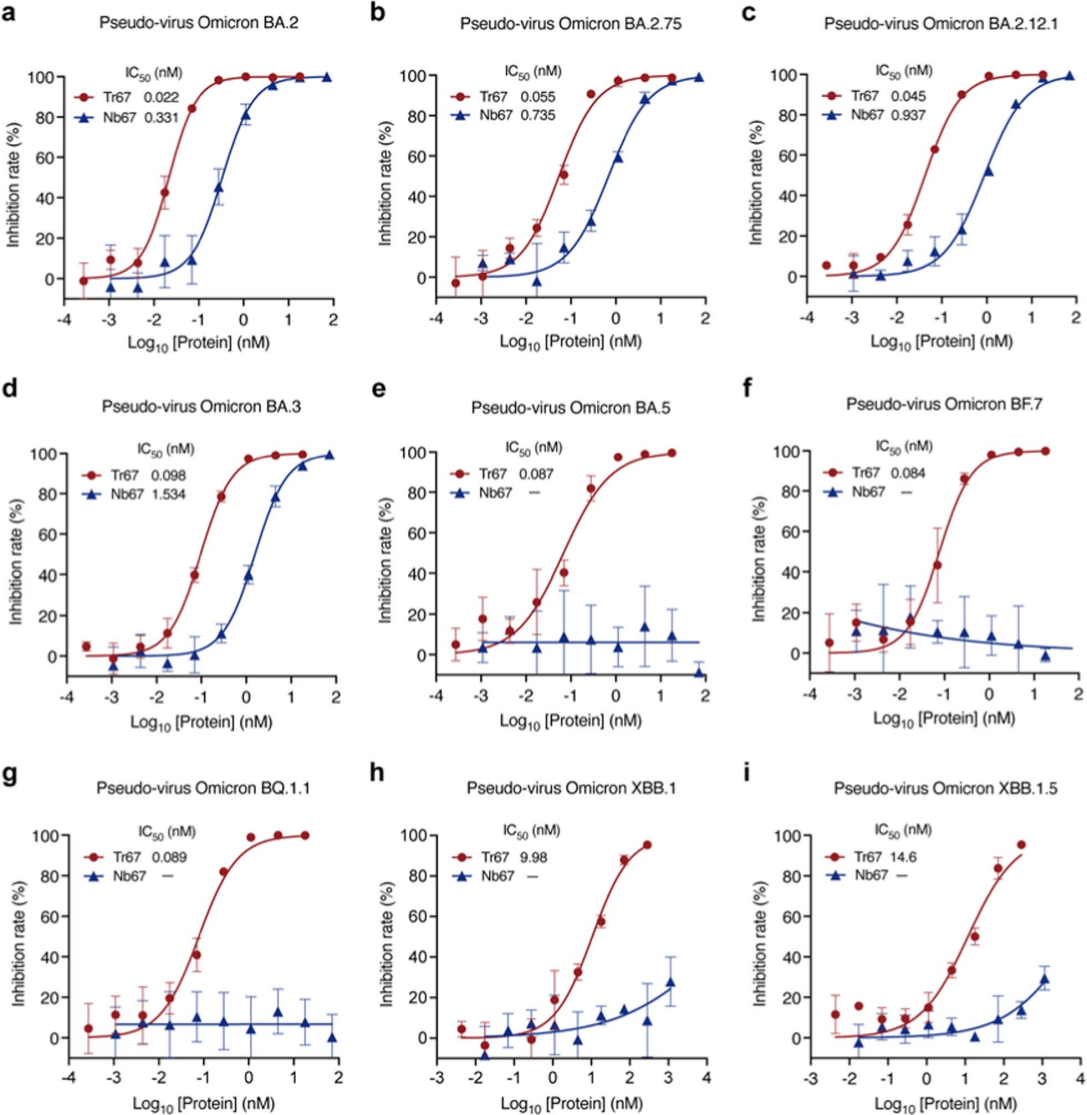
Broad-spectrum neutralization potential of Tr67 against the dominant SARS-CoV-2 Omicron variants. **a**—**i** Neutralizing activities against SARS-CoV-2 pseudoviruses Omicron BA.2, BA.2.75, BA.2.12.1, BA.3, BA.5, BF.7, BQ.1.1, XBB.1, and XBB.1.5 variants, respectively. Three independent experiments were performed for each variant

To further investigate the broad-spectrum neutralizing potential of Tr67, we evaluated its neutralization activities against the dominant Omicron variants (Fig. 7). For Omicron BA.2, Tr67 exhibited an IC_50_ of 0.022 nM and that of Nb67 is 0.331 nM, so the neutralizing activity was greatly enhanced by about 15 folds (Fig. 7a). Similar enhancements were observed for Omicron BA.2.75, BA.2.12.1, and BA.3: the corresponding IC_50_ values of Tr67 were 0.055, 0.045, and 0.098 nM, respectively, and those of Nb67 were 0.735, 0.937, and 1.534 nM, respectively (Figs. 7b–d). Unexpectedly, Tr67 also neutralized the variants that are more likely to evade humoral immunity. For Omicron BA.5, BF.7, and BQ.1.1, Nb67 failed to achieve any detectable neutralization; however, Tr67 neutralized them with IC_50_ values of 0.087, 0.084, and 0.089 nM, respectively (Figs.7e–g). Even for the most immune-evasive Omicron XBB family, Tr67 still maintained neutralizing activity, but Nb67 did not (Figs.7h, i). Specifically, the IC_50_ values of Tr67 against XBB.1 and XBB.1.5 were 9.98 and 14.6 nM, respectively. Thus, compared with its monovalent counterpart, Tr67 has a significant increase in the neutralizing activity against all the tested Omicron variants, suggesting that multivalent proteins have the potential to be developed into broad-spectrum drugs against the emerging SARS-CoV-2 variants.

### Cryo-EM analysis of Tr67-spike complex

**Fig. 8:**
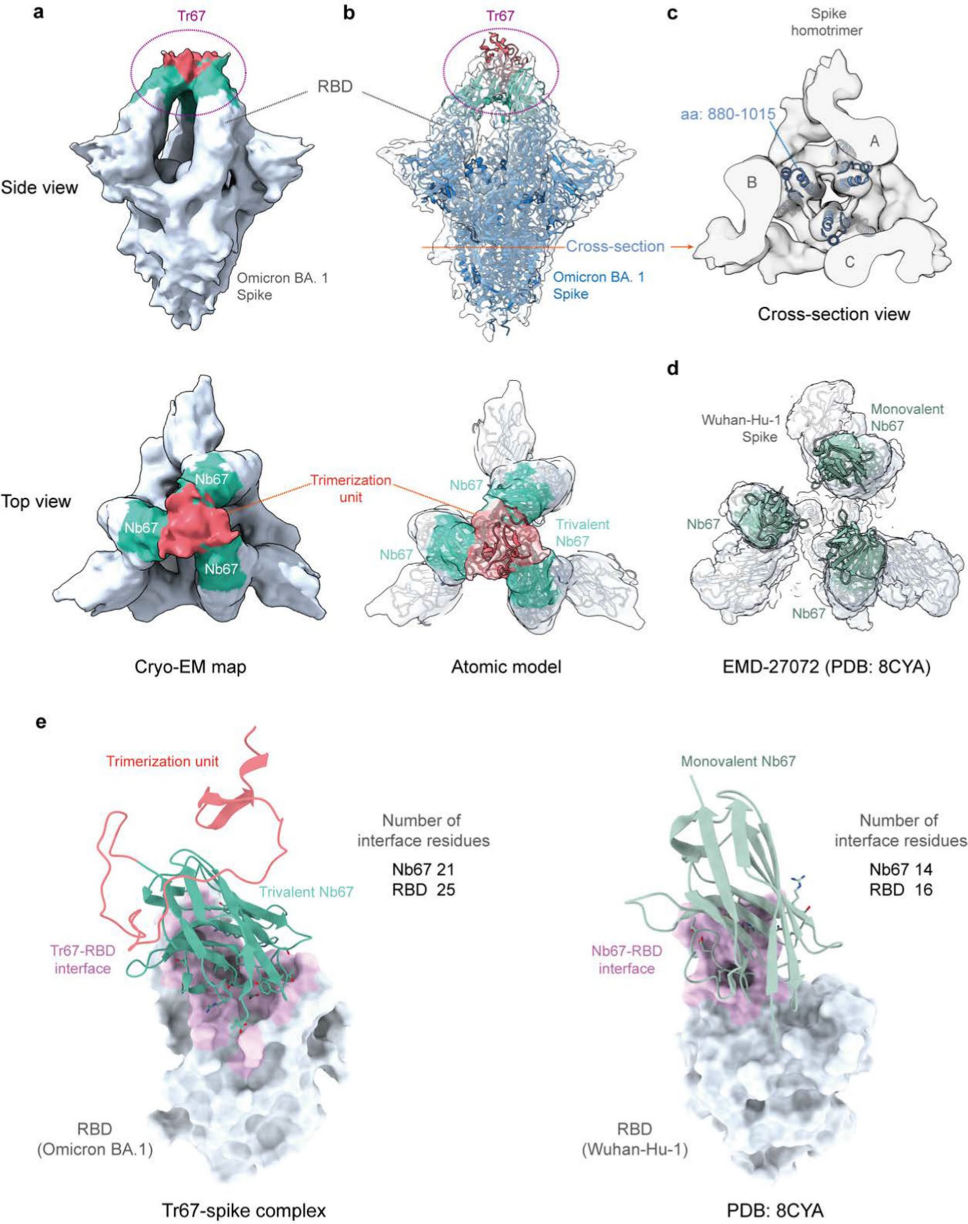
Cryo-EM structures of Tr67 in complex with the SARS-CoV-2 (Omicron BA.1) spike protein. **a** EM density map of Tr67-spike complex in “3-RBD-up” conformation at the overall resolution of 9 Å. **b** Fitting of the atomic model of the designed Tr67-spike complex into the EM density map. The EM density is shown as a transparent gray surface and the spike protein is rendered in blue. **c** Cross-section view of the stem region of the spike protein. **d** An open-like, “3-RBD-up” conformation with the SARS-CoV-2 (Wuhan-Hu-1) spike protein induced by three separated Nb67. **e** The binding interfaces of trivalent (left panel) and monovalent (right panel) Nb67 binding with the RBDs (shown as gray surface) are shown in pink, and the contact residues on Nb67 are shown as sticks

Finally, to confirm whether the binding mode of Tr67 to the spike protein is consistent with our design, we determined the complex structure of Tr67 with the Omicron BA.1 spike protein using cryo-EM ((Additional file 1: Figs. S4, Table S3 and Fig. 8). As seen in Fig. 8A, the cryo-EM density map obtained by the single-particle 3D reconstruction method clearly shows that the complex structure is a triple-symmetric homotrimer; moreover, the density of Tr67 bound to the RBDs at the top of the S-protein is very well defined (Fig. 8a, side view), and the density of the three Nb67 nanobodies and the trimerization unit 5ff (Fig. 8a, top view) can also be distinguished. Therefore, the cryo-EM structure provided experimental evidence that Tr67 is indeed bound to the epitopes specified by the computational design.

To obtain the atomic model of the cryo-EM structure, we used the MD flexible fitting method to fit the atomic model of the Tr67-spike complex constructed in the computational design into the density map (Fig. 8b). As can be seen, the atomic model of the whole complex fitted well into the map; and this became clearer by the illustration of the central alpha-helical regions in the S protein stem (Fig. 8c). More specifically, the designed Tr67 matches well with corresponding densities, showing that Tr67 exactly binds to the desired positions on the S protein (Fig. 8b, top view).

Significantly, we found that the complex structure is one in which all 3 RBDs of the S protein are in the standing-up state (3-RBD-up). Due to its amino acid mutations and RBD-RBD interactions, the Omicron spike is usually stabilized in the “2-RBD-down, 1-RBD-up” conformation; this conformational state was considered to facilitate the up-RBD to approach ACE2 and then to promote membrane fusion [36–38]. Unlike the spikes of early variants such as Wuhan-Hu-1, the Omicron spike rarely occurs in the 3-RBD-up conformation, which may need to be induced by a combination of distinct antibodies [39, 40]. Thus, the cryo-EM structure did confirm that Tr67 can induce the Omicron spike into the 3-RBD-up conformation. Note that, unlike the common 3-RBD-up conformation (Wuhan-Hu-1) induced by monovalent nanobodies, in which the three RBDs are in an open-like, unassociated state (Fig. 8d), Tr67 has an additional trimerization unit that covalently links the three binders and thereby firmly locks the three RBDs in an inactive state (Fig. 8b, top view). Unsurprisingly, the S protein in such a Tr67-bound state cannot bind ACE2 anymore and therefore its membrane fusion function is completely inhibited.

To understand the molecular basis of the increased binding affinity of Tr67 for the S protein, we analyzed the binding interfaces of monovalent Nb67 and Tr67 with the RBDs using the Nb67-spike structure and the atomic model of the Tr67-spike complex. We identified the interface (contact) residues by a 4-Å distance cutoff between the atoms of Nb67 and those of the RBD, as shown in Fig. 8e and listed in (Additional file 1: Table S4. As can be seen, the binding sites of Nb67 and Tr67 on the RBDs are identical to those of ACE2. However, the Nb67 binder in Tr67 has a larger contact area with the Omicron RBD, and the interface contains 21 residues from Nb67 and 25 residues from the Omicron RBD. In contrast, the monovalent Nb67 and the Wuhan Hu-1 RBD have only 14 and 16 interfacial residues, respectively. In addition, the Nb67 binder in Tr67 forms more hydrogen bonds and salt bridges (Additional file 1: Table S4), suggesting stronger binding interactions. Consistent with the BLI results in Fig. 6c, the number of interfacial residues also supports that Tr67 could establish a more extensive network of interactions, contributing to stronger binding to the Omicron BA.1 and thus enhancing its neutralizing activity.

To explain why Tr67 also binds to other Omicron variants (Additional file 1: Fig. S5), we built structural models for their RBDs based on the atomic models in Fig. 8b, and then analyzed their binding interfaces with the monovalent Nb67 and Tr67 by docking simulation using PyDock [41]. The best-scoring binding poses from the simulations were used as the representatives of the Nb67-spike and Tr67-spike complexes, as shown in (Additional file 1: Figs. S6 and S7, respectively. As illustrated in (Additional file 1: Fig. S6a, for the variants BA.1, BA.2, BA.2.75, BA.2.12.1, and BA.3 (cluster 1), Nb67 was successfully docked into the expected epitope on RBD; however, for variants BA.5, BF.7, BQ.1.1, XBB.1, and XBB.1.5 variants (cluster 2), Nb67 in the best-scoring poses was not located at the expected sites, but at other sites that are sterically unfavorable in the 1-RBD-up conformation of the S protein (Additional file 1: Fig. S6b). Consistent with this, Nb67 was able to neutralize the Omicron variants in cluster 1 but not those in cluster 2 (Fig. 7). Obviously, the amino-acid mutations of the cluster 2 variants weaken the interactions of the monovalent Nb67 with the variant RBD sites in Fig. 8e and thus abolish the neutralization. Particularly, similar to a previous study [35], we found that the mutation at 486 plays a key role in this process (Additional file 1: Fig. S5 and Table S5). In contrast, for all variants, Tr67 of the best-scoring poses binds to the same epitopes as that of BA.1. The binding interfaces are also larger than those of Nb67 (Additional file 1: Table S6). It appears that the synergistic binding of the three Nb67s in Tr67 increases the binding interface and then leads to higher binding affinities that could resist the mutations such as that at 486 to some extent. As a result, Tr67 is still able to bind to the same epitopes of the Omicron BA.1 S protein and neutralize the other variants tested. Clearly, further structural studies are needed to elucidate the detailed molecular mechanisms involved.

## Discussion

In this study, we developed a computational approach for engineering nanoscale multivalent protein drugs and successfully designed two highly potent anti-SARS-CoV-2 trivalent microproteins: MP-5ff and Tr67. The trimerization unit designed by our method enabled these two proteins to efficiently self-assemble and achieve good conformational homogeneity. Clearly, the multivalency of the proteins increased their binding affinities and thus enhanced their virus-neutralizing ability. In particular, Tr67 was able to neutralize dominant Omicron variants, including the extensively drug-resistant XBB.1 and XBB.1.5, and thus has the potential to be developed as a broad-spectrum anti-SARS-CoV-2 drug. Moreover, the cryo-EM structure confirmed that Tr67 stably binds to all three RBDs of the S protein in a synergistic manner, consistent with the designed binding mode.

As confirmed by the cryo-EM structure, our computational approach enabled a precise design and rational engineering of multivalent proteins that geometrically match the binding sites of a given target protein. Different from many previous studies of engineering multivalent proteins, our approach incorporates structure-guided modeling and MD evaluation to predict the potential effects of various fusion formats of trimerization scaffolds and linkers on the binding geometry and self-assembly properties. Compared with experimental screening that may require more resources and time to identify suitable trimerization scaffolds or fusion linkers [18, 42, 43], our rational approach can narrow the design space and reduce randomness in screening potential multivalent proteins against SARS-CoV-2. No doubt, this is important for accelerating the development of multivalent therapeutics and providing a timely response to the rapidly evolving SARS-CoV-2.

Very interestingly, we captured the structure of the Omicron BA.1 spike protein in the 3-RBD-up conformation, and thus provided mechanistic insights into the antiviral activity of the trivalent nanobody Tr67. As reported, the Omicron spikes exclusively adopt a “1-RBD-up, 2-RBD-down” conformation [44, 45]; even in the presence of ACE2 or neutralizing antibodies, the transition to a “3-RBD-up” conformation was rarely observed for the Omicron variants [38, 46]. Previous studies have showed that this conformation could be induced via the combination of two antibodies targeting distinct epitopes on a single RBD [39, 40]. Interestingly, Tr67 achieved this by synergistically interacting with the same epitope on the three RBDs. In this sense, the induced mechanism of the 3-RBD-up conformation by Tr67 is different from the previous one. To the best of our knowledge, our cryo-EM structure is the first 3-RBD-up conformation induced by such a mechanism. Also, the “1-RBD-up, 2-RBD-down” conformation was considered to give the Omicron variants an advantage in evading the immune system, because many antibodies fail to bind their epitopes when two of the RBDs are in the “down” state [38, 45]. Instead, the “3-RBD-up” conformation exposes those epitopes and thereby facilitates more effective immune recognition and clearance. Therefore, by inducing the 3-RBD-up conformation, Tr67 not only blocks the ACE2 binding to any RBD of the S protein, but also leads the S protein to a conformational state more susceptible to immune clearance.

Again, our study clearly shows that multivalent microproteins like Tr67 are ideal candidates for antiviral drugs. These proteins are well suited for large-scale production in *E. coli* due to their small size, structural stability, and solubility. They have higher target-binding affinity and better virus-neutralizing activity than their monovalent counterparts, and are also more resistant to viral escape. Indeed, Tr67 effectively neutralized a range of Omicron variants, including XBB.1 and XBB.1.5, unlike most neutralizing antibodies, which lose potency against these variants [5, 47, 48]. As shown in Fig. 8a, the neutralizing activity Tr67 can be attributed to its unique multivalent structure, which allows synergistic engagement of all three RBDs, thereby inducing the spike 3-RBD-up conformation. Such multivalent binding results in the so-called binding avidity [49] − the accumulated binding strength derived from the three binders − is much greater than that of a single, monovalent Nb67 binding to S by inducing the “1-RBD-up, 2-RBD-down” conformation. This not only results in wider binding interfaces (e.g., in Fig. 8e), but also ensures that all three bound binders (Nb67s) in Tr67 remain tightly associated with the RBDs. For example, even if one bound binder temporarily dissociates from RBD, the other two binders will bring it back into the bound state.

In addition to the designed protein assemblies in this study, some recent studies have also explored various nanomaterials as vaccines and drugs against SARS-CoV-2, including the RBD-conjugated lung-derived exosome vaccine [50], liposomal-based nanotraps with ACE2 or neutralizing antibodies on the surface [51], cell membrane-derived ACE2-containing nanocatchers [52], lung spheroid cell (LSC)-mimicking nanodecoys displaying ACE2 [53], nanosponges made of human-cell-derived membranes with receptors (e.g., ACE2) [54], and 2D nanosheets of graphene oxide (GO) [55] and CuInP_2_S_6_ [56]. Together with ours, these studies demonstrated that the design and use of nanoscale biomolecular/matter assemblies is a very effective way to combat SARS-CoV-2 infection. Similar to the ACE2 protein on nanotraps, nanocathers, LSC-nanodecoys, and nanosponges, our multivalent proteins are also specific binders to the spike RBDs of SARS-CoV-2 S, and thus might serve as the neutralizing antibodies on the surfaces of these nanomaterials, such as on nanotraps and nanocatchers. As a proof of concept, our study mainly focused on the computational design of a series of self-assembling multivalent microproteins and the in vitro validation of their physicochemical properties, improved binding affinity, and neutralizing activity by comparison with the monovalent counterparts. In the future, further research is needed to investigate the in vivo therapeutic efficacy, pharmacokinetics, immunogenicity, and biosafety of Tr67 consisting of the foldon domain, GS-linker and nanobody. In the past, the foldon domain has been used in several protein vaccines [28, 29, 57]. Meanwhile, at least three nanobody drugs have been approved for clinical use, namely Caplacizumab [58], Ozoralizumab [59, 60], and Envafolimab [61]. Among them, Ozoralizumab also used a similar GS-linker to connect its three nanobodies in a tandem format. Anyway, comprehensive in vivo evaluations of all these components are crucial for the translation of Tr67 into clinical applications.

In summary, our study has successfully established a computational approach for designing multivalent antiviral microproteins. Using this method, we have engineered nanoscale trivalent microproteins with therapeutic potential against SARS-CoV-2. Especially, the trivalent nanobody Tr67 displayed favorable physicochemical properties and showed strong neutralizing activity against the dominant Omicron variants, demonstrating its potential for further development as a broad-spectrum anti-SARS-CoV-2 drug. Furthermore, this study provides an effective strategy applicable for designing and engineering nanoscale multivalent drugs targeting other disease proteins, such as S in MERS-CoV, HA in influenza, Env in HIV, F protein in RSV, and TNF-α in cancer.

## Materials and methods

### Structure-guided design of linkers

RosettaRemodel [62] was used to design linkers connecting the C-terminus of miniACE2 and the N-terminus of the trimerization scaffold. This program samples backbone conformations by incorporating fragments randomly selected from a database of known protein structures. Fragment insertion was guided by Ramachandran restraints and clash avoidance. A blueprint file was prepared to specify the desired connectivity, secondary structure, and sequence of the linker regions, and the “0 × L” entries in the blueprint file allowed backbone flexibility for fragment insertion. The linker sequence was also specified in the blueprint file and fragment sampling was restricted to match it. We tested (GGGGS)_n_ and (EAAAK)_n_ linkers with n = 2, 3, 4, and 5, respectively; and, the “-no_jumps” flag was used to control the folding process within the linker, enabling sampling of degrees of freedom in the connecting region and determining the optimal structures. For each linker candidate, 1000 independent trajectories were sampled, and the lowest-energy models from these trajectories were then saved as PDB files. All the molecular graphics were generated by UCSF ChimeraX [63].

### All-atom MD simulation

The initial three-dimensional (3D) structures of the trivalent constructs were modeled using SWISS-MODEL [64]. All-atom MD simulations were performed using GROMACS software with AMBER 99SB-ILDN force field [65, 66]. The proteins were solvated in a cubic box with the SPC water model and neutralized with Na^+^ and Cl^−^ ions. Energy minimization was performed using the steepest descent algorithm for 5000 steps to remove atomic clashes. The Particle-Mesh-Ewald (PME) [67] algorithm was used to calculate long-range electrostatic interactions; a cut-off of 1.4 nm was used for short-range interactions and van der Waals forces. Covalent bonds involving hydrogen atoms were constrained using the LINCS algorithm [68]. The system was equilibrated by a 100-ps NVT simulation at 300 K using the velocity rescaling thermostat [69], followed by 100-ps NPT equilibration at the pressure of 1 bar using the Berendsen barostat [70]. For each construct, three independent 300-ns production runs were performed.

### Free energy landscape analysis

Free energy landscapes (FELs) map the possible conformations of a given protein and their associated energy levels in the MD simulation [71]. FEL visualizes the conformational energy function versus the given configuration space, which usually is a two-dimensional space. To construct the FELs, principal component analysis (PCA) was first performed on alpha carbon (Cα) coordinates of a given simulated protein. The gmx covar tool in GROMACS was used to calculate the covariance matrix, which was then diagonalized to obtain eigenvectors and eigenvalues. Next, we used the gmx anaeig tool to project the simulated conformations onto the first two principal components (PC1 and PC2), which accounted for the most variance. For each simulated trajectory, the R studio and Bio3D package [72] was used to calculate the cumulative variance rate of PC1 and PC2; and for each construct, the trajectory with the highest cumulative variance contribution was selected as the representative. Accordingly, the Gibbs free energy of a given conformation at the two-dimensional space defined by PC1 (*x*) and PC2 (*y*) was calculated using the following equation:

1 $$\Delta G=-{k}_{B}T ln\frac{p\left(x,y\right)}{{p}_{max}}$$

where ${k}_{B}$ is Boltzmann constant, $T$ is the simulation temperature, and $p\left(x,y\right)$ is the probability distribution along the two given reaction coordinates, *x* and *y*. ${p}_{max}$ is the maximal probability of the distribution. In addition to the FELs built by PCA, we also constructed FELs using root mean square deviation (RMSD) and radius of gyration (Rg) as the projection coordinates, which provide a complementary perspective on the conformational space defined by PC1 and PC2. The Matplotlib package in Python was used to visualize the FELs.

### MM/GBSA calculation

The gmx_MMPBSA package was used to calculate the binding free energy of the monomers of the trivalent proteins via the MM/GBSA (Molecular Mechanics/Generalized–Born Surface Area) method [73, 74]. And 1500 frames were extracted from the last 150 ns of each trajectory for analysis. The AMBER ff99SB-ILDN force field was used to determine the internal term (E_int_), van der Waals (E_vdW_), and electrostatic (E_ele_) energies. The modified Generalized Born model GB^OBC1^ (igb = 2) was used to estimate the polar contribution of the solvation energy (GGB), while the nonpolar energy is estimated using the solvent-accessible surface area (SASA). Here, the self-assembling binding free energy of the monomers was computed as:

2 $$\Delta \Delta {G}_{bind}=\Delta {G}_{complex}-\Delta {G}_{A}-\Delta {G}_{B}-\Delta {G}_{C}$$

where A, B, and C represent the three monomers (chains) of the trivalent protein, respectively; $\Delta {G}_{A}$ is obtained by calculating the binding free energy between monomer A and the complex unit BC; and $\Delta {G}_{B}$ and $\Delta {G}_{C}$ were obtained in the same way. The mean and standard deviation of $\Delta \Delta {G}_{bind}$ were calculated by averaging over the three independent simulations for each system.

### Protein expression and purification

To construct the trivalent constructs using miniACE2, linker sequences were inserted after miniACE2, followed by the trimerization scaffolds. Codon-optimized genes encoding the designed protein sequences were cloned into a modified pET-29b (+) vector with an N-terminal 8 × His-tag and TEV cleavage site. The plasmids were transformed into *E. coli* Rosetta (DE3) cells. The cells were cultured at 37 ℃ in LB broth until an OD_600_ of ~ 0.6–0.8 was reached, then induced with IPTG at 20 ℃ overnight. Cells were then harvested, sonicated, and lysed on ice with lysis buffer (pH 7.4, 1 × PBS, 20 mM Imidazole). The soluble fraction was extracted by centrifugation at 12,000 rpm for 30 min. For purification of the proteins, cell supernatants were filtered through a 0.45 μm syringe and applied to Ni–NTA gravity-flow columns pre-equilibrated with buffer A (pH 7.4, 1 × PBS, 20 mM Imidazole). The columns were washed sequentially with buffer A and 1 × PBS containing 80 mM Imidazole before eluting the His-tag proteins with buffer B (pH 7.4, 1 × PBS, 300 mM Imidazole). The proteins were then analyzed by sodium dodecyl sulfate–polyacrylamide gel electrophoresis (SDS-PAGE).

For the nanobody Nb67 and its trivalent construct Tr67, codon-optimized genes for Nb67 (with an N-terminal PelB sequence for periplasmic secretion and a C-terminal 6 × His-tag) and Tr67 (with an N-terminal 6 × His-tag and TEV cleavage site) were cloned into pET-26b (+). The plasmids were transformed into *E. coli* Rosetta (DE3) cells. Nb67 expression was induced at 25 ℃ for 20 h. Periplasmic extracts were obtained by osmotic shock. Expression steps for Tr67 were consistent with those for the miniACE2-based trivalent constructs, and purification also followed the same protocol mentioned above.

To remove His-tags, purified proteins were desalted using a HiTrap desalting column and then incubated overnight at 4 ℃ with TEV protease. Tag-free proteins were further isolated by Ni–NTA chromatography.

### Size exclusion chromatography

A Superdex 200 Increase 10/300 GL column attached to the ÄKTA avant (GE Healthcare) system was used for size exclusion chromatography (SEC) to detect and separate the oligomeric populations of the purified trivalent proteins. The column was equilibrated with 1 × PBS, pH 7.4. Then, 500 µL of concentrated trivalent protein was loaded onto the column. Proteins were eluted at a flow rate of 0.5 mL/min, and the protein signals were monitored by measuring the ultraviolet light absorbance at 280 nm. The contents of different oligomeric fractions were evaluated by the peak integration area. The trimeric fractions were collected for further characterization.

### Native polyacrylamide gel electrophoresis

The trimeric fractions isolated from SEC were analyzed by Native polyacrylamide gel electrophoresis (Native-PAGE) to evaluate their conformational homogeneity [75]. The protein samples were diluted in native sample loading buffer and subjected to 15% native gels. Electrophoresis was conducted using a native running buffer at 120 V for approximately 1.5 h at 4 ℃. Gels were subsequently stained to visualize the trimer protein bands.

### Biolayer interferometry measurement

The binding kinetics of the trivalent constructs and corresponding monomers to the RBD of S protein were determined by Biolayer interferometry (BLI) using an Octet RED96e system (Sartorius/ForteBio). Briefly, each protein was diluted in running buffer (1 × PBS, 0.02% Tween-20, 0.1% BSA) and transferred to a 96-well plate. For miniACE2 and MP-5ff assay, the RBD (Wuhan-Hu-1) was immobilized onto HIS1K biosensors (ForteBio) following the manufacturer’s protocol. For Nb67 and Tr67 assay, RBD (Omicron BA.1) was immobilized onto streptavidin (SA) biosensors (Sartorius/ForteBio). After equilibrating in the running buffer, the sensors with immobilized RBD were dipped into wells containing the protein sample at various concentrations (50–3.125 nM for miniACE2; 100–3.125 nM for MP-5ff; 500–31.25 nM for Nb67; 100–3.125 nM for Tr67) for association 1 min, followed by dissociation for 3 min. Binding curves were fitted by a 1:1 binding model with the Octet Data Analysis software (ForteBio). The *k*_on_, *k*_off_, and K_D_ values were then determined from curves with R^2^ > 0.95.

### Pseudovirus neutralization assay

The SARS-CoV-2 pseudoviruses carrying firefly luciferase reporter gene were generated with vesicular stomatitis virus (VSV) pseudotyping system according to a published protocol [76]. Briefly, plasmids encoding prototype or variant SARS-CoV-2 spike proteins (including Wuhan-Hu-1, Omicron BA.1, BA.2, BA.2.75, BA.2.12.1, BA.3, BA.5, BF.7, BQ.1.1, XBB.1 and XBB.1.5, respectively) were transfected into HEK 293 T cells together with VSVΔG pseudovirus particles. At 24 h post-infection, the viral supernatants were harvested, centrifuged, and filtered to remove cell debris, then stored at − 80 ℃. Pseudovirus titers were calculated by testing the median tissue culture infective dose (TCID_50_) with a human ACE2 overexpression cell line [76]. For neutralization assays, the pseudoviruses were diluted to ~ 10^4^ TCID_50_/mL in cell culture medium. Then, 75 μL protein sample dilution was mixed with 25 μL pseudovirus dilution in a 96-well white flat bottom plate, and incubated at 37 ℃ for 1 h. After incubation, 100 μL of human ACE2 overexpression HEK 293 cell suspension were added to each well (5 × 10^4^ cells/well). After incubation at 37 ℃, 5% CO_2_ (v/v) for 24 h, infection was quantified by luminescence measurement using a luminescence meter (PerkinElmer, Cat. No. HH34000000). The inhibition ratio was calculated with the following formula:

3 $$Inhibition\; ratio=\left(1-\frac{{\text{X}}-\overline{CC} }{\overline{VC }-\overline{CC} }\right)\times 100\%$$

where *X* is the luminescence value (RLU) of a certain well; CC is the cell control with only cells are added; $\overline{{\text{CC}} }$ is the mean value of cell control group; VC is the virus control with only cells and pseudovirus are added; $\overline{{\text{VC}} }$ is the mean value of virus control group. Each assay was done in triplicate. Data were fitted using non-linear regression, and the IC_50_ values were calculated using a four-parameter regression equation in GraphPad Prism software.

### Single-particle cryo-EM analysis

The Tr67-Omicron BA.1 spike complex was prepared by manual mixture of the two proteins in a 1.5:1 weight ratio, then diluted to a final concentration of 0.3 mg ml^−1^. Samples (2 μL) were applied to a 200 mesh 1.2/1.3R Cu Quantifoil grid and vitrified using a Vitrobot Mark IV with a blot time of 6.5 s in the environment of 23 ℃ and 100% humidity before the grid was plunged into liquid ethane.

All cryo-EM data were collected on a 200 kV FEI Glacios Cryo Transmission Electron Microscope (Thermo Fisher Scientific) equipped with a FEI Falcon 3EC direct detector. Movie stacks were collected automatically using the EPU software (Thermo Fisher Scientific) at a magnification of 92,000 × with a pixel size of 1.57 Å. Each movie stack of Tr67-Omicron BA.1 spike complexes with 40 frames was exposed under a total dose of 43 electrons per Å^2^. The defocus range was set from − 1.8 to − 2.4 μm. In total, 4969 videos were acquired for the single particle analysis.

As illustrated in (Additional file 1: Fig. S4, for single particle analysis, the frames of each image stack (skipping the first and last frame) were motion-corrected with RELION [77] implementation of the MotionCor2 [78] algorithm and the corrected micrographs were imported into cryoSPARC [79] for further processing. Contrast transfer function (CTF) value estimations were performed by Patch CTF Estimation. Particle coordinates were automatically picked by PARSED [80] with diameters from 100 to 300 Å, and the 5,250,976 particles were extracted with a box size of 280 pixels and a pixel size of 1.57 Å. Several rounds of iterative 2D classifications were then performed to select well-defined particle images, and about 254,339 particles were selected for further 3D reconstruction. Three parallel ab initio reconstructions were conducted in cryoSPARC and a total of 144,101 particles contributing to the spike-like density were selected. These particles were subjected to one round of homogeneous refinement with C3 symmetry, and eventually generated an EM density map at the resolution of ~ 9 Å.

### Supplementary Information

**Additional file 1.** **Figure S1.** Time-dependent RMSDs of the trivalent constructs. **Figure S2.** Principal component analysis (PCA) for a MD trajectory of MP-5ff. **Figure S3.** The free energy landscapes (FELs) of the MD conformations of Tr67. **Figure S4.** Flowchart for the single-particle cyro-EM analysis. **Figure S5.** RBD mutations in the Omicron variants tested. **Figure S6.** Best-scoring PyDock docking poses of monovalent Nb67 to the RBDs of the Omicron variants. **Figure S7.** Best-scoring PyDock docking poses of Tr67 to the RBDs of the Omicron variants. **Table S1.** The amino acid sequences of the trivalent constructs. **Table S2.** MM/GBSA binding free energies of trivalent constructs in MD simulations. **Table S3.** Cryo-EM data collection and refinement statistics. **Table S4.** Interfacial residues of monovalent Nb67 and Tr67 binding to RBD. **Table S5.** Interfacial residues of the RBDs of the cluster 1 variants with the monovalent Nb67. **Table S6.** Binding interface areas and numbers of interfacial residues of the best-scoring docking poses of Nb67 and Tr67 to the RBDs of the tested Omicron variants.

## Data Availability

All data generated or analyzed during this study have been included in the article and the Additional information. The cryo-EM density map of Tr67 in complex with the spike protein of Omicron BA.1 has been deposited in the Electron Microscopy Data Bank with accession code EMD-36850.
