# Supplementary material for: Computational design and engineering of self-assembling multivalent microproteins with therapeutic potential against SARS-CoV-2
Source: J Nanobiotechnology. 2024 Feb 10;22:58. doi: 10.1186/s12951-024-02329-3 (PMC10858482; doi:10.1186/s12951-024-02329-3)
Supplement: Supplementary file 1 — Additional file 1. Figure S1. Time-dependent RMSDs of the trivalent constructs. Figure S2. Principal component analysis (PCA) for a MD trajectory of MP-5ff. Figure S3. The free energy landscapes (FELs) of the MD conformations of Tr67. Figure S4. Flowchart for the single-particle cyro-EM analysis. Figure S5. RBD mutations in the Omicron variants tested. Figure S6. Best-scoring PyDock docking poses of monovalent Nb67 to the RBDs of the Omicron variants. Figure S7. Best-scoring PyDock docking poses of Tr67 to the RBDs of the Omicron variants. Table S1. The amino acid sequences of the trivalent constructs. Table S2. MM/GBSA binding free energies of trivalent constructs in MD simulations. Table S3. Cryo-EM data collection and refinement statistics. Table S4. Interfacial residues of monovalent Nb67 and Tr67 binding to RBD. Table S5. Interfacial residues of the RBDs of the cluster 1 variants with the monovalent Nb67. Table S6. Binding interface areas and numbers of interfacial residues of the best-scoring docking poses of Nb67 and Tr67 to the RBDs of the tested Omicron variants. [file 12951_2024_2329_MOESM1_ESM.pdf]

**Additonal file 1**

# Computational design and engineering of self-assembling multivalent microproteins with therapeutic potential against SARS-CoV-2

Qin Qin[1], Xinyi Jiang[1], Liyun Huo[1], Jiaqiang Qian[1], Hongyuan Yu[2], Haixia Zhu[1], Wenhao Du[1], Yuhui Cao[2], Xing Zhang[2], and Qiang Huang[1, 3] *

[1]*State Key Laboratory of Genetic Engineering, Shanghai Engineering Research Center of Industrial Microorganisms, MOE Engineering Research Center of Gene Technology, School of Life Sciences, Fudan University, Shanghai 200438, China*
[2]*ACROBiosystems Inc., Beijing 100176, China*
[3]*Multiscale Research Institute of Complex Systems, Fudan University, Shanghai 201203, China*

*Corresponding author at: School of Life Sciences, Fudan University, Shanghai 200438, China.
Email: huangqiang@fudan.edu.cn

*List of Supplementary Materials:*

Figure S1. Time-dependent RMSDs of the trivalent constructs.

Figure S2. Principal component analysis (PCA) for a MD trajectory of MP-5ff.

Figure S3. The free energy landscapes (FELs) of the MD conformations of Tr67.

Figure S4. Flowchart for the single-particle cyro-EM analysis.

Figure S5. RBD mutations in the Omicron variants tested.

Figure S6. Best-scoring PyDock docking poses of monovalent Nb67 to the RBDs of the Omicron variants.

Figure S7. Best-scoring PyDock docking poses of Tr67 to the RBDs of the Omicron variants.

Table S1. The amino acid sequences of the trivalent constructs.

Table S2. MM/GBSA binding free energies of trivalent constructs in MD simulations.

Table S3. Cryo-EM data collection and refinement statistics.

Table S4. Interfacial residues of monovalent Nb67 and Tr67 binding to RBD.

Table S5. Interfacial residues of the RBDs of the cluster 1 variants with the monovalent Nb67.

Table S6. Binding interface areas and numbers of interfacial residues of the best-scoring docking poses of Nb67 and Tr67 to the RBDs of the tested Omicron variants.

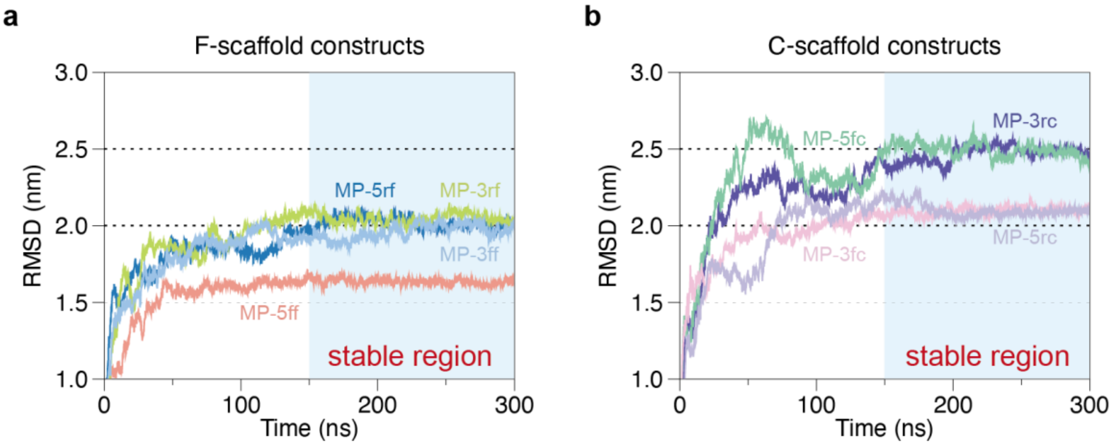


**Figure S1.** Time-dependent RMSDs of the trivalent constructs averaged over three independent MD trajectories, with their initial structures as the references. (a) The RMSD results of the four F-scaffold constructs. (b) The RMSD results of the four C-scaffold constructs.

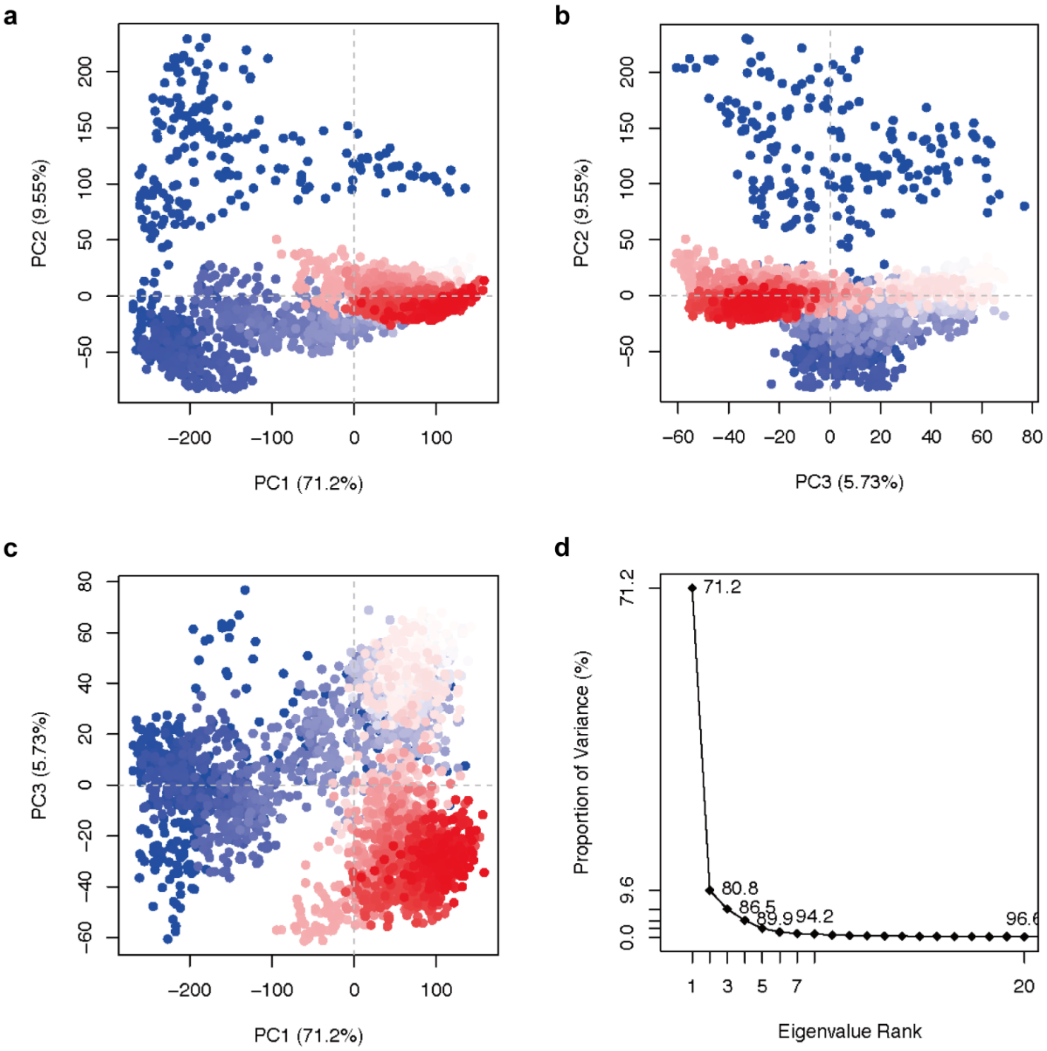


**Figure S2.** Principal component analysis (PCA) for a MD trajectory of the trivalent construct MP-5ff. (a) Projection of the trajectory onto the first and the second principal components (PC1 and PC2). (b) Projection of the trajectory onto the second and the third principal components (PC2 and PC3). (c) Projection of the trajectory onto the first and the third principal components (PC1 and PC3). (d) Corresponding eigenvalue contributions of the principal components to the variance of the data.

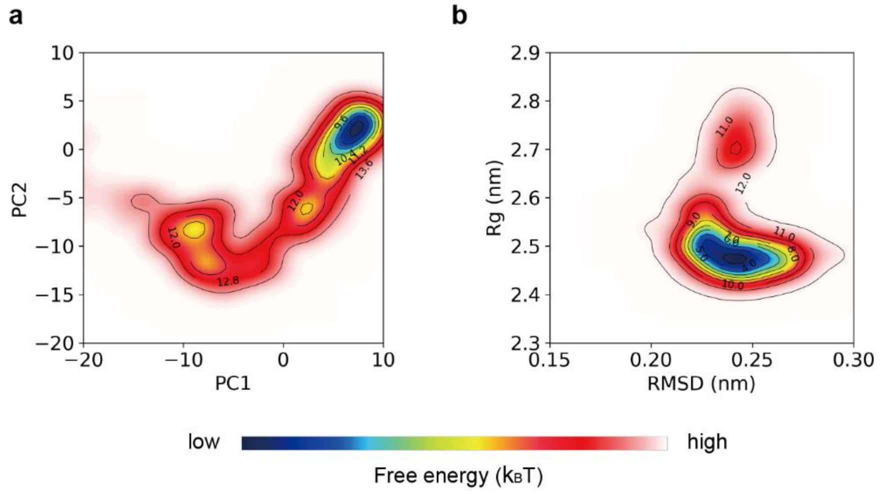


**Figure S3.** The free energy landscapes (FELs) of the MD conformations of the trivalent nanobody Tr67. (a) FEL for the simulated conformational projections onto the first and the second principal components (PC1 and PC2), indicating that there exists only a deep free-energy well (in blue). (b) FEL for the simulated conformational projections onto two alternative reaction coordinates: root mean square deviation (RMSD) and radius of gyration (Rg), showing again that there exists only a deep free-energy well (in blue). Both FELs showed that Tr67 has only one low-energy trimer conformation, suggesting a good conformational homogeneity similar to that of MP-5ff.

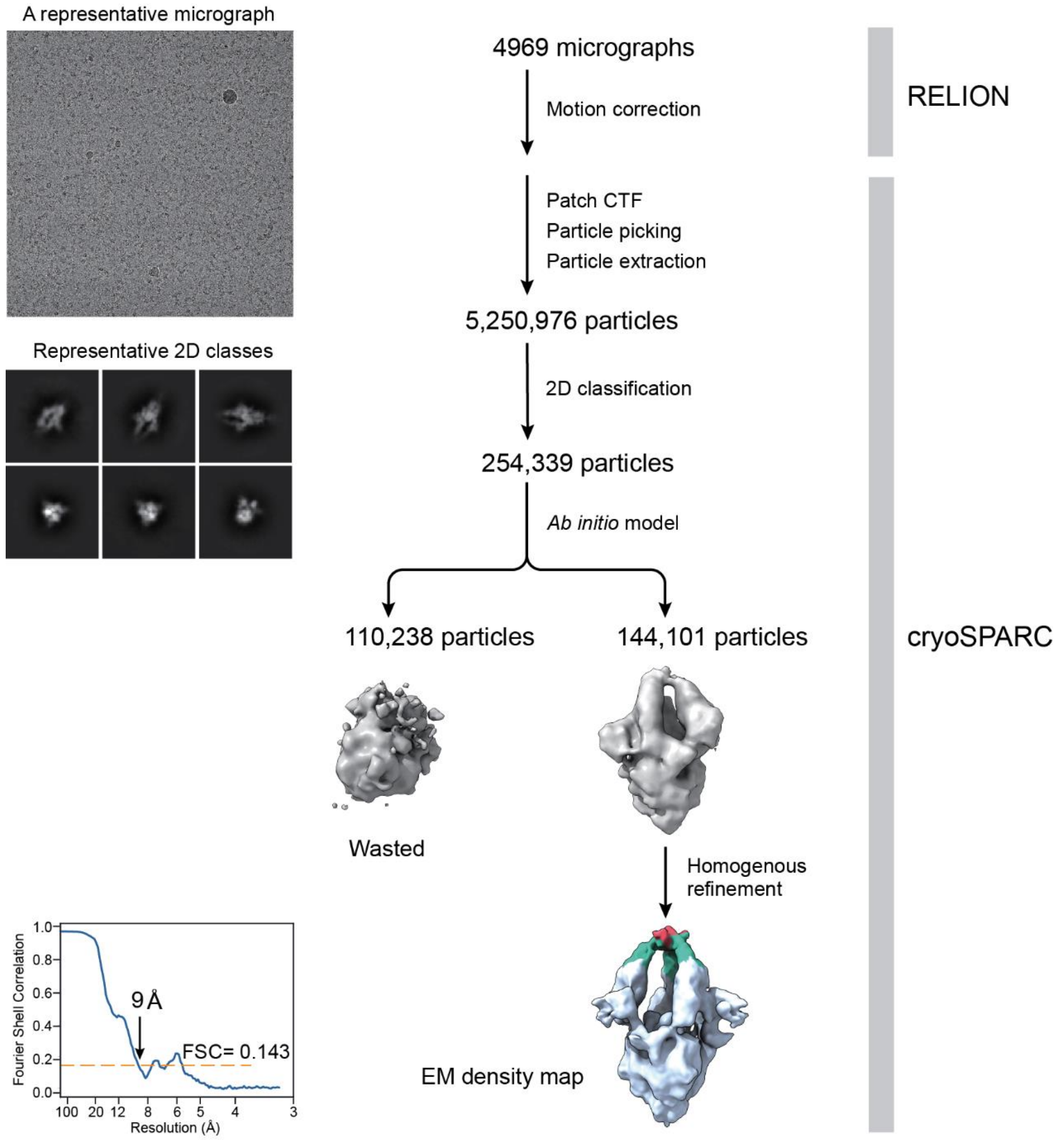


**Figure S4.** Flowchart for the single-particle cyro-EM analysis of Tr67 in complex with Omicron BA.1 spike protein. The overall resolution of the EM density map is ~9 Å with the Fourier shell correlation (FSC) at 0.143.

| | 339 | 346 | 368 | 371 | 373 | 375 | 376 | 405 | 408 | 417 | 440 | 444 | 445 | 446 | 452 | 460 | 477 | 478 | 484 | 486 | 490 | 493 | 496 | 498 | 501 | 505 |
|---|---|---|---|---|---|---|---|---|---|---|---|---|---|---|---|---|---|---|---|---|---|---|---|---|---|---|
| **BA.1** | D | R | L | L | P | F | T | D | R | N | K | K | V | S | L | N | N | K | A | F | F | R | S | R | Y | H |
| **BA.2** | | | | F | | | A | N | S | | | | | G | | | | | | | | | G | | | |
| **BA.2.75** | H | | | F | | | A | N | S | | | | | | | K | | | | | | Q | G | | | |
| **BA.2.12.1** | | | | F | | | A | N | S | | | | | G | Q | | | | | | | | G | | | |
| **BA.3** | | | | F | | | | N | | | | | | | | | | | | | | | G | | | |
| **BA.4/5** | | | | F | | | A | N | S | | | | | G | R | | | | | V | | Q | G | | | |
| **BF.7** | | T | | F | | | A | N | S | | | | | G | R | | | | | V | | Q | G | | | |
| **BQ.1.1** | | T | | F | | | A | N | S | | | T | | G | R | K | | | | V | | Q | G | | | |
| **XBB.1** | H | T | I | F | | | A | N | S | | | | P | | | K | | | | S | S | Q | G | | | |
| **XBB.1.5** | H | T | I | F | | | A | N | S | | | | P | | | K | | | | P | S | Q | G | | | |

**Figure S5.** RBD mutations in the Omicron variants tested. Blank positions indicate residues conserved relative to BA.1, and colored positions highlight residues with mutations different from the BA.1 sequence. Structural models of the RBDs of the variants were built using the BA.1 atomic model as the template and the RosettaRomodel program[62]. A total of 500 low-energy models were generated for each variant, and the lowest-energy model was selected as the input structure for molecular docking.

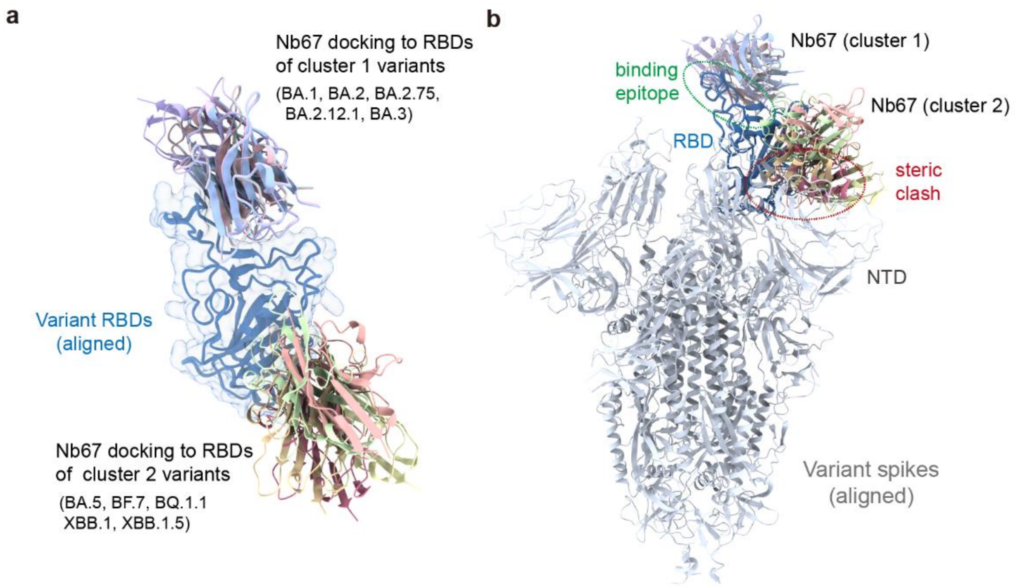


**Figure S6**. Best-scoring PyDock docking poses of monovalent Nb67 to the RBDs of the Omicron variants. (a) Nb67 docked to the expected epitope on the upper region of the RBD for variants BA.1, BA.2, BA.2.75, BA.2.12.1, and BA.3 (cluster 1), but to the lower region for variants BA.5, BF.7, BQ.1.1, XBB.1, and XBB.1.5 (cluster 2). (b) The docking Nb67-RBD complexes in the S proteins with the 1-RBD-up conformation. The Nb67s of the cluster 2 variants might collide with other parts of the S protein, suggesting that they are sterically unfavorable for effective binding to the RBDs. The PyDOCK server at https://life.bsc.es/pid/pydock was used to perform the docking simulations, and PyDOCK scoring is based on an empirical potential composed of electrostatic, desolvation, and van der Waals energy terms.

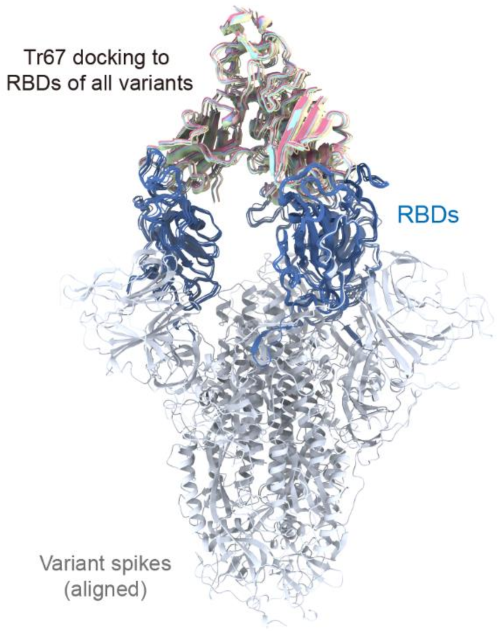


**Figure S7.** Best-scoring PyDock docking poses of Tr67 to the RBDs of the Omicron variants. The docking Tr67-spike complexes in the superimposed S proteins with the 3-RBD-up conformation. The PyDOCK server at https://life.bsc.es/pid/pydock was used to perform the docking simulations.

**Table S1.** The amino acid sequences of the trivalent constructs.

| Name | Monomer sequence |
|---|---|
| MP-3ff | NDDELHMLMTDLVYEALHFAKDEEIKKRVFQLFELADKAYKNNDRQKLEKVVEELKELLERLLSGGGGSGG GGSGGGGSGYIPEAPRDGQAYVRKDGEWVLLSTFL |
| MP-3rf | NDDELHMLMTDLVYEALHFAKDEEIKKRVFQLFELADKAYKNNDRQKLEKVVEELKELLERLLSEAAAKEA AAKEAAAKGYIPEAPRDGQAYVRKDGEWVLLSTFL |
| MP-5ff | NDDELHMLMTDLVYEALHFAKDEEIKKRVFQLFELADKAYKNNDRQKLEKVVEELKELLERLLSGGGGSGG GGSGGGGSGGGGSGGGGSGYIPEAPRDGQAYVRKDGEWVLLSTFL |
| MP-5rf | NDDELHMLMTDLVYEALHFAKDEEIKKRVFQLFELADKAYKNNDRQKLEKVVEELKELLERLLSEAAAKEA AAKEAAAKEAAAKEAAAKGYIPEAPRDGQAYVRKDGEWVLLSTFL |
| MP-3fc | NDDELHMLMTDLVYEALHFAKDEEIKKRVFQLFELADKAYKNNDRQKLEKVVEELKELLERLLSGGGGSGG GGSGGGGSGEIAALKQEIAALKKEIAALKFEIAALKQGYY |
| MP-5fc | NDDELHMLMTDLVYEALHFAKDEEIKKRVFQLFELADKAYKNNDRQKLEKVVEELKELLERLLSGGGGSGG GGSGGGGSGGGGSGGGGSGEIAALKQEIAALKKEIAALKFEIAALKQGYY |
| MP-3rc | NDDELHMLMTDLVYEALHFAKDEEIKKRVFQLFELADKAYKNNDRQKLEKVVEELKELLERLLSEAAAKEA AAKEAAAKGEIAALKQEIAALKKEIAALKFEIAALKQGYY |
| MP-5rc | NDDELHMLMTDLVYEALHFAKDEEIKKRVFQLFELADKAYKNNDRQKLEKVVEELKELLERLLSEAAAKEA AAKEAAAKEAAAKEAAAKGEIAALKQEIAALKKEIAALKFEIAALKQGYY |
| Tr67 | EVQLVESGGGLVQTGGSLRLSCALSGYTFSIFPTAWFRQAPGKEREFVAGIRWNGSTRDYTEYADFVKGRF TISRDNAKNMVYLQMISLKPEDTALYYCAASDGVIDGTNANAYRYWGQGTQVTVSSGGGGSGGGGSGGGGS GGGGSGGGGSGYIPEAPRDGQAYVRKDGEWVLLSTFL |

**Table S2.** MM/GBSA binding free energies of trivalent constructs in MD simulations.

| | Trivalent constructs | Trajectory No. | Energies (kcal·mol$^{-1}$) | Mean values (kcal·mol$^{-1}$) | Standard deviations |
|---|---|---|---|---|---|
| F-scaffold trimers | MP-3rf | 1 | -243.29 | -243.3 | 10.39 |
| | | 2 | -232.92 | | |
| | | 3 | -253.70 | | |
| | MP-5rf | 1 | -246.17 | -224.6 | 19.86 |
| | | 2 | -220.67 | | |
| | | 3 | -207.05 | | |
| | MP-3ff | 1 | -246.08 | -235.4 | 27.17 |
| | | 2 | -204.52 | | |
| | | 3 | -255.62 | | |
| | MP-5ff | 1 | -272.36 | -288.6 | 15.81 |
| | | 2 | -303.94 | | |
| | | 3 | -289.43 | | |
| C-scaffold trimers | MP-3rc | 1 | -164.08 | -172.1 | 6.96 |
| | | 2 | -175.91 | | |
| | | 3 | -176.35 | | |
| | MP-5rc | 1 | -246.07 | -248.2 | 12.36 |
| | | 2 | -261.44 | | |
| | | 3 | -236.98 | | |
| | MP-3fc | 1 | -166.84 | -164.2 | 12.20 |
| | | 2 | -150.87 | | |
| | | 3 | -174.83 | | |
| | MP-5fc | 1 | -216.07 | -222.4 | 6.18 |
| | | 2 | -228.43 | | |
| | | 3 | -222.65 | | |

**Table S3.** Cryo-EM data collection and refinement statistics

| | Tr67-spike (Omicron BA.1) complex |
|---|---|
| **Data collection and processing** | |
| Microscope | FEI Glacios with Falcon 3 direct detector |
| Magnification | 92,000 |
| Voltage (kV) | 200 |
| Electron exposure ($e^{-}$/Å$^2$) | 43 |
| Frame exposure ($e^{-}$/Å$^2$) | 1.075 |
| Defocus range (μm) | -1.8 to -2.4 |
| Pixel size (Å) | 1.57 |
| Total micrographs | 4969 |
| | |
| **3D reconstruction** | |
| Auto-picked particles | 5,250,976 |
| Particles in final refinement | 144,101 |
| Symmetry imposed | C3 |
| Final resolution (Å) | 9.0 |
| FSC threshold | 0.143 |

**Table S4.** Interfacial residues of monovalent Nb67 and Tr67 binding to RBD.[a]

| Nb67-spike structure (PDB:8CYA) | | Atomic model of Tr67-spike complex | |
|---|---|---|---|
| Monovalent Nb67 | RBD (Wuhan-Hu-1) | Trivalent Nb67 | RBD (Omicron BA.1) |
| P33 | L455 | E1 | R408 |
| R52 | F456 | G26 | T415 |
| N54 | K458 | Y27 | G416 |
| S56 | Y473 | T28 | N417 |
| T57 | A475 | S30 | D420 |
| R58 | G476 | I31 | Y421 |
| Y60 | S477 | F32 | Y453 |
| E62 | V483 | P33 | R454 |
| G104 | E484 | R52 | L455 |
| V105 | G485 | W53 | F456 |
| I106 | F486 | N54 | K458 |
| D107 | N487 | S102 | Y473 |
| G108 | C488 | D103 | N477 |
| T109 | Y489 | G104 | Y489 |
| | F490 | V105 | P491 |
| | Q493 | I106 | L492 |
| | | D107 | R493 |
| | | T109 | S494 |
| | | N110 | S496 |
| | | A113 | F497 |
| | | R115 | R498- |
| | | | P499 |
| | | | T500 |
| | | | Y501 |
| | | | H505 |

[a] Interfacial residues were identified by a 4-Å distance cutoff between the atoms of Nb67 and those of RBD. Residues involved in hydrogen bonding are highlighted in red and those forming salt bridges are highlighted in yellow.

**Table S5.** Interfacial residues of the RBDs of the cluster 1 variants with the monovalent Nb67.[b]

| Variants | Interfacial residues of RBD |
|---|---|
| BA.1 | 449, 455, 456, 475, 483, 484, 485, 486, 487, 488, 489, 490, 493, 494 |
| BA.2 | 449, 455, 456, 475, 484, 485, 486, 487, 488, 489, 490, 492, 493, 494 |
| BA.2.75 | 449, 483, 484, 485, 486, 489, 490, 492, 493, 494 |
| BA.2.12.1 | 449, 452, 455, 456, 475, 484, 485, 486, 487, 488, 489, 490, 492, 493, 494 |
| BA.3 | 449, 455, 475, 483, 484, 485, 486, 487, 488, 489, 490, 493, 494 |

[b]The mutation at 486 was found to be the key residue for the binding to cluster 1 variants, distinguishing them from cluster 2 variants BA.5, BF.7, BQ.1.1, XBB.1, and XBB.1.5 (as shown in Fig. S5).

**Table S6.** Binding interface areas and numbers of interfacial residues of the best-scoring docking poses of Nb67 and Tr67 to the RBDs of the tested Omicron variants.[c]

| Variants | Nb67 docking to single RBD | | Tr67 docking to RBDs | |
|---|---|---|---|---|
| | Interface area ($Å^2$) | Number of RBD residues | Interface area ($Å^2$) | Number of RBD residues |
| BA.2 | 733.7 | 14 | 1005.1 | 22 |
| BA.2.75 | 555.9 | 10 | 1024.3 | 19 |
| BA.2.12.1 | 734.4 | 15 | 942.3 | 18 |
| BA.3 | 707.9 | 13 | 1031.6 | 21 |
| BA.5 | – | – | 945.9 | 20 |
| BF.7 | – | – | 948.3 | 23 |
| BQ.1.1 | – | – | 1001.4 | 20 |
| XBB.1 | – | – | 760.2 | 15 |
| XBB.1.5 | – | – | 738.2 | 14 |

[c] The interface areas and residue numbers of both the Nb67-RBD and Tr67-RBD complexes were calculated based on the binding interactions with a single RBD.
